# NHE7-Driven endoplasmic reticulum stress signaling transmission promotes malignant progression of endometrial cancer via c-Fos/PRSS1/ERP27 axis

**DOI:** 10.1186/s13046-026-03773-9

**Published:** 2026-07-01

**Authors:** Shizhou Yang, Yuejiang Ma, Zhengyun Chen, Tingting Wu, Xiufeng Huang

**Affiliations:** 1 Department of Gynecology, Women’s Hospital, Zhejiang University School of Medicine, 1# Xueshi Road, Shangcheng District, Hangzhou, Zhejiang China; 2 Zhejiang Key Laboratory of Precision Diagnosis and Therapy for Major Gynecological Diseases, Hangzhou, Zhejiang China

**Keywords:** Endometrial Cancer, NHE7, PRSS1, Endoplasmic reticulum stress, C-Fos, ERP27

## Abstract

**Background:**

Endometrial cancer (EC) threatens women’s health. The Na⁺/H⁺ Exchanger 7 (NHE7) is implicated in cancer progression, but its role in EC and potential link to endoplasmic reticulum (ER) stress remain poorly understood.

**Methods:**

NHE7 was overexpressed or knocked down in EC cells. Functional assays and transcriptomic analyses were performed. Supernatant (SN) or exosomes from donor EC cells were applied to recipient cells. Mechanistic studies included exosome inhibition (GW4869), bioinformatic analysis, dual-luciferase reporter assays, chromatin immunoprecipitation (ChIP), and ER stress manipulation (4-PBA/Tunicamycin). In vivo tumor models validated findings.

**Results:**

NHE7 promoted malignant phenotypes, EMT, and stemness via ER stress activation. SN/exosomes from NHE7-overexpressing cells transmitted these phenotypes and ER stress to recipient cells, which were blocked by GW4869 or 4-PBA. PRSS1 was identified as a key NHE7 downstream target, and its exosomal delivery mediated ER stress transmission. Mechanistically, NHE7 drove PRSS1 transcription in donor cells via ER stress-dependent c-Fos recruitment to the PRSS1 promoter. In recipient cells, PRSS1 upregulated ERP27 to amplify ER stress. TCGA data showed that high PRSS1 expression correlated with poor prognosis. In vivo, NHE7 accelerated tumor growth and upregulated PRSS1/ERP27, effects reversed by 4-PBA.

**Conclusion:**

NHE7 functions as a key regulator of ER stress signaling transmission in EC cells, driving malignant phenotypes and stemness through the c-Fos/PRSS1/ERP27 axis. Targeting this axis may offer a novel therapeutic strategy for EC.

**Graphical Abstract:**

**Figure:**
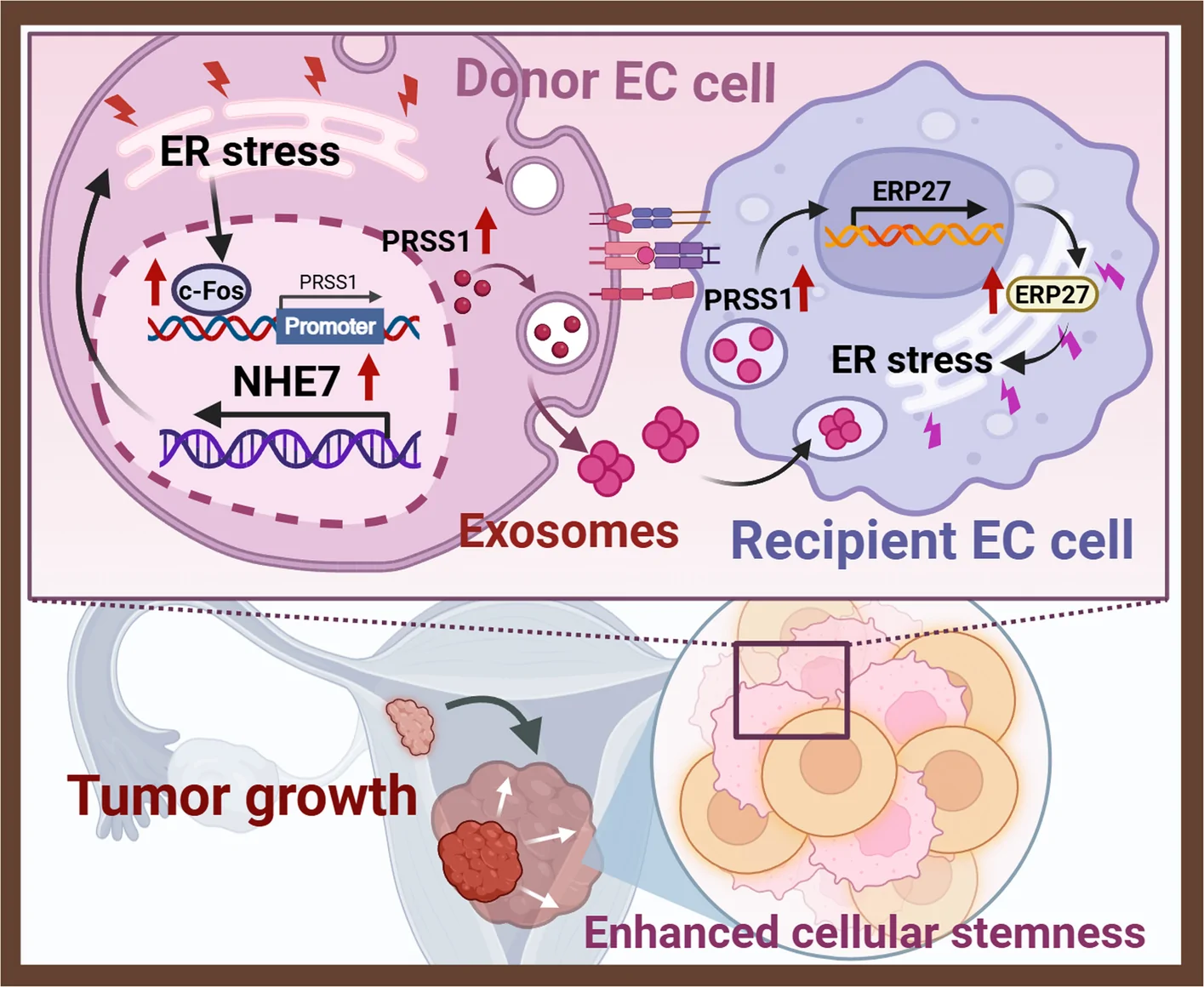

**Supplementary Information:**

The online version contains supplementary material available at https://doi.org/10.1186/s13046-026-03773-9.

## Introduction

Endometrial cancer (EC) has exhibited a steady increase in incidence and mortality globally over the past few decades, emerging as a significant threat to women's health [1]. Although there have been refinements in operative techniques and adjuvant treatments, patients with advanced or recurrent EC still face low 5-year survival probabilities, largely attributable to the complex interplay of genetic, epigenetic, and tumor microenvironmental factors [2]. Many underlying mechanisms remain poorly understood, highlighting the critical need for in-depth exploration of novel molecular targets and signaling pathways.

The Na^+^/H^+^ Exchanger 7 (NHE7), a member of the Solute Carrier Family 9 (SLC9A) family of ion transporters, is primarily localized to the trans-Golgi network and endosomal membranes, where it plays a pivotal role in regulating intracellular pH homeostasis and vesicular trafficking dynamics [3]. Emerging evidence has established the association between NHE7 dysregulation and oncogenesis across multiple malignancies. In hepatocellular carcinoma (HCC), for instance, NHE7 overexpression enhances the maturation of macropinosomes, thereby promoting the uptake of small extracellular vesicles (sEVs) secreted by HCC cells. These tumorigenic sEVs mediate the transfer of oncogenic cargo that promotes neoplastic proliferation, migratory capacity, and chemoresistance [4]. Pancreatic cancer studies reveal NHE7's essential role in maintaining Golgi apparatus acidity and cytoplasmic alkalinity, which are critical for cancer cell metabolism and survival, with its inhibition shown to induce tumor regression [5]. In breast cancer, overexpression of NHE7 enhances cell adhesion, invasion, and in vivo tumorigenesis, contributing to disease progression [6]. Notably, NHE7 has been identified as a clinically significant prognostic biomarker in EC, with immunohistochemical studies confirming its substantial overexpression in malignant tissues. Our previous study demonstrated that NHE7 promotes tumor progression by simultaneously enhancing cancer cell proliferation and migration while inhibiting cellular senescence through a cAMP-dependent signaling pathway that involves Ca^2+^ influx [7]. These multifaceted oncogenic properties position NHE7 as a potential therapeutic target for EC treatment.

Endoplasmic reticulum (ER) stress, a conserved cellular response to perturbations in protein folding and calcium homeostasis, has been established as a key contributor to tumorigenesis and cancer progression [8]. In the context of EC, the tumor microenvironment, characterized by hypoxia, nutrient deprivation, and oxidative stress, frequently triggers chronic ER stress, which activates the unfolded protein response (UPR) [9]. The UPR, mediated by three primary sensors, protein kinase RNA-like endoplasmic reticulum kinase (PERK), inositol-requiring enzyme 1 (IRE1), and activating transcription factor 6 (ATF6), aims to restore ER homeostasis. However, in the long term, sustained UPR activation can promote tumor cell survival, angiogenesis, and immune evasion [10]. For example, glucose-regulated protein 78 (GRP78), a central chaperone molecule in the ER stress response, is often overexpressed in EC tissues, and its high levels correlate with advanced tumor stage and poorer patient survival [11]. Notably, cellular FBJ osteosarcoma viral oncogene homolog (c-Fos), a core component of the activator protein-1 (AP-1) transcription complex, has been implicated in UPR signaling [12, 13]. Emerging evidence directly connects c-Fos-mediated UPR adaptation to EC progression, suggesting its potential as a therapeutic target [14]. Endoplasmic reticulum protein 27 (ERP27), a non-catalytic member of the protein disulfide isomerase family with a hydrophobic pocket for unfolded protein binding, participates in ER stress regulation by collaborating with endoplasmic reticulum protein 57 (ERP57) to facilitate protein folding [15]. Its dysregulation in cancers like breast and colorectal cancer has been linked to UPR-mediated therapeutic resistance [16, 17]. These findings highlight the critical role of ER stress signaling in EC pathogenesis and suggest that targeting this pathway may offer therapeutic potential.

Although previous studies have identified NHE7 as a significant contributor to cancer progression, and ER stress is recognized as a pivotal factor in EC pathogenesis, the potential functional relationship between NHE7 and ER stress signaling in EC remains incompletely understood. In this study, we hypothesized that NHE7 promotes EC malignancy by activating ER stress signaling cascades, and that this activation enables intercellular transmission of pro-tumorigenic signals to recipient cells. To test this hypothesis, we first characterized the expression pattern of NHE7 across EC cell lines and performed bidirectional gain- and loss-of-function studies to establish its oncogenic role. We then investigated whether NHE7-activated ER stress signals could be transmitted to recipient cells and explored the downstream molecular mechanisms. This study aims to elucidate a novel NHE7-centered mechanism in EC pathogenesis and to identify potential therapeutic targets for this malignancy.

## Materials and methods

### Cell culture and drug treatment

Ishikawa cells (YDT-0302) were cultured in Dulbecco's Modified Eagle Medium (DMEM, BDBIO, Beijing, China, L100-500), and HEC-1-A cells (YDT-0225) were maintained in McCoy's 5 A Medium (Pricella, Wuhan, China, PM150710). KLE cells (YDT-0327) were cultured in DMEM/F12 medium (BDBIO, Beijing, China, L310-500), RL95-2 cells (YDT-0565) were maintained in DMEM/F12 medium supplemented with 0.005 mg/mL insulin (Sigma-Aldrich, USA, I9278) and 0.5% (v/v) ITS (Insulin-Transferrin-Selenium, Thermo Fisher, USA, 41400045), and AN3 CA cells (YDT-0059) were cultured in DMEM (BDBIO, Beijing, China, L100-500). All media were supplemented with 10% fetal bovine serum (FBS, SERANA, China, s-mx-015) and 1% penicillin/streptomycin (P/S, Gibco, USA, 15140–122). The cell lines were all obtained from INDIT Bio-Technology Co., Ltd. (Hangzhou, China).

In the mechanism verification experiment, cells were treated with 4-Phenylbutyric acid (4-PBA, 10 μM, Selleck Chemicals, USA, S3592), an ER stress inhibitor, for 24 h in complete medium (37℃, 5% CO_2_). For exosome inhibition, cells were treated with GW4869 (10 μM, MedChemExpress, USA, HY-19363) for 24 h to block exosome release. For ER stress induction, cells were treated with Tunicamycin (10 μg/mL, MedChemExpress, USA, HY-A0098) for 24 h. For the Serine Protease 1 (PRSS1, 100 ng/mL, CUSABIO, CSB-EPO18811HUI) recombinant protein treatment, Ishikawa and HEC-1-A cells (5 × 10^5^ cells/well in 6-well plates) were treated with the protein for 24 h.

### Cell transfection

siR-PRSS1 and siR-NHE7 constructs (Hippo Bio, China) were transfected into 70–80% confluent EC cells in 6-well plates. The transfection complex was prepared by mixing 2 μL siRNA (100 nM final concentration) and 2 μL Lipofectamine 2000 (Invitrogen) in Opti-MEM I Reduced Serum Medium (Gibco), with the sequences provided in Table 1.

**Table 1:** The sequences of siRNAs

Name	Sense	Antisense
siRNA-PRSS-1	GCUCUCCUCACGUGCAGUAAUTT	AUUACUGCACGUGAGGAGAGCTT
siRNA-PRSS-2	AGGUCUACAACUAUGUGAAAUTT	AUUUCACAUAGUUGUAGACCUTT
siRNA-PRSS-3	AGAACACCAUAGCUGCCAAUATT	UAUUGGCAGCUAUGGUGUUCUTT
siRNA-NHE7	CUUGGAUCUAUACUGGCCUAU	AUAGGCCAGUAUAGAUCCAAG

For overexpression plasmid transfections, 6 μg of pcDNA3.1-NHE7 or 6 μg of empty pcDNA3.1 vector (Youbio, Hunan, China) was combined with 2 μL of Lipofectamine 2000, followed by 24 h of incubation with cells before assessment. Puromycin was used to screen for Ishikawa cells stably overexpressing NHE7.

### Transwell assay

Cell migration was assessed using 8 μm Transwell inserts (Corning, 3422). EC cells (2 × 10^4^ cells/well) in serum-free medium were added into the upper chamber, while the lower chamber was filled with the culture medium containing 10% FBS. After 24 h, non-migrated cells were removed, and migrated cells were fixed with methanol (20 min; Sinopharm, 10014108) and stained with 0.1% crystal violet (Aladdin, C110703, 40 min). For invasion assays, inserts were pre-coated with Matrigel (30 min; Corning, 356234, 37 °C). Quantification was performed by counting stained cells in five random microscopic fields per well.

### Methylthiazolyl diphenyltetrazolium bromide (MTT) assay

For MTT assay, drug-treated EC cells (3 × 10^3^ cells/well) were seeded in 96-well plates. After culturing for designed time points (0–72 h), 50 μL MTT solution (Sigma, M2128) was added per well and incubated for 3 h at 37 °C. The culture medium was then carefully removed, and 150 μL DMSO (HUSHI, 30072418) was added to each well to dissolve the formazan crystals. The absorbance was measured at 570 nm (Thermofisher, 51119570).

### Sphere formation assay

To evaluate cancer stem cell-like properties, EC cells (3 × 10^4^ cells/well) were seeded in ultra-low attachment 6-well plates (Corning, USA, 3471). Cells were cultured in serum-free DMEM/F12 medium (BI, Germany, 01–172-1ACS) supplemented with 5 μg/mL insulin (Prospec, Israel, CYT-27), 20 ng/mL epidermal growth factor (EGF, Prospec, CYT-217), 10 ng/mL fibroblast growth factor (FGF, Prospec, CYT-218), and B27 supplement at a final concentration of 1 × (Gibco, MA, USA, 12587010). The cells were cultured for two weeks, during which spheres were monitored daily. After culture, spheres were manually counted under a microscope across the entire well. Spheres were defined as non-adherent, three-dimensional cell aggregates with a diameter greater than 75 μm. Images were captured from multiple random fields per well using a microscope, and representative images are shown. The experiment was performed independently three times, with three technical replicates per condition in each experiment. Counting was performed in a blinded manner, where the observer was unaware of the treatment group assignments.

### Bioinformatics

Transcriptome sequencing data were analyzed. Differential expression analysis was performed using the limma package (v3.62.2) or DESeq2 (v1.46.0) in R(cutoffs: *p* < 0.05, |log2FC|> 0.26). Visualization was achieved through volcano plots generated by ggplot2. Functional enrichment analysis of these differentially expressed genes (DEGs) was subsequently conducted for Gene Ontology (GO) terms and Kyoto Encyclopedia of Genes and Genomes (KEGG) pathways using the clusterProfiler package (v4.14.6).

To investigate ER stress-related mechanisms, the ER stress-related gene set was obtained from the GeneCards database (https://www.genecards.org). A Venn diagram was subsequently generated using the R package ggvenn (v0.1.16) to visualize the overlap between these DEGs and the ER stress-related gene set.

The RNA-seq data and corresponding clinical information of EC were downloaded from the TCGA database, comprising a total of 585 samples (550 tumor tissues and 35 normal controls). For differential gene expression analysis, the tumor samples were stratified into PRSS1 high- and low-expression groups based on the median expression level of PRSS1. Differential gene expression analysis was performed using DESeq2 (v1.46.0), with significant DEGs defined as those with an absolute log2 fold change greater than 1 and a *p*-value below 0.05. The DEGs were visualized in a volcano plot generated by ggplot2 (v3.5.1). To assess the prognostic significance of PRSS1, patients with PRSS1 expression levels above the optimal cutoff value were classified as the high-expression group, while those with expression levels below the cutoff were classified as the low-expression group, and survival analysis was performed using the R packages survival (v3.7–0) and survminer (v0.5.0). To compare PRSS1 expression between tumor and adjacent normal tissues, the Wilcoxon rank-sum test was used due to the imbalance in sample sizes between the two groups. This non-parametric method does not assume equal sample sizes or normal distribution and is robust against outliers, making it appropriate for TCGA-based analyses. The results were visualized through box plots generated using the ggpubr (v0.6.0) and ggplot2 (v3.5.1) packages in R. The correlation between PRSS1 and SLC9A7 was evaluated by constructing scatter plots with the ggpubr package (v0.6.0).

The ER stress-related gene set was obtained from the GeneCards database (https://www.genecards.org) using a Relevance score cutoff greater than 4.5, which selected 3,327 genes. Transcription factors regulating PRSS1 were acquired from hTFtarget (https://guolab.wchscu.cn/hTFtarget/#!/). Venn analysis was performed using the R package ggvenn (v0.1.16) to identify the intersection between the upregulated differentially expressed genes (log2 fold change > 0.5, *p*-value < 0.05) and the ER stress-related gene set. Survival analysis was performed using TCGA endometrial tumor samples, with patients divided into high- and low-expression groups based on the optimal cutoff value for each gene, using the R packages survival (v3.7–0) and survminer (v0.5.0).

### Western blotting (WB) analysis

WB analysis was conducted to examine protein expression levels. Cellular or tissue samples were lysed in RIPA buffer followed by centrifugation (14,000 × g, 10 min, 4 °C) to collect supernatants. Protein quantification was performed using the BCA method (Thermo, USA, 23228). After loading equal protein quantities (20 μg) onto SDS-PAGE gels (8–12% gradient), separated proteins were transferred to PVDF membranes (Millipore, IPVH00010). Membranes were blocked with 5% bovine serum albumin (BSA, Solarbio, A8020) in TBST (Tris-buffered saline with 0.1% Tween-20) for 1 h at room temperature before overnight incubation with primary antibodies at 4 °C. Following three TBST washes, HRP-conjugated secondary antibodies were applied for 1 h at room temperature. Signal detection was achieved using ECL reagent (Biosharp, BL520B) and captured with a chemiluminescence imaging system (SH-523). Antibody specifications are provided in Table 2, with β-actin serving as internal control. Triplicate biological replicates were performed for all experiments.

**Table 2:** The information on antibodies

Name	Brand	No	Dilution rate	WB/IHC
E-cadherin	proteitech	20874–1-AP	1:20000	WB
N-cadherin	CST	13116	1:1000	WB
Vimentin	CST	5741	1:1000	WB
β-actin	proteintech	20536–1-ap	1:4000	WB
p-PERK	Invitrogen	MA5-15033	1:1000	WB
PERK	CST	5683 T	1:1000	WB
p-IRE1α	Invitrogen	PA1-16927	1:500	WB
IRE1α	CST	3294 T	1:1000	WB
ATF6	proteintech	24169–1-AP	1:2000	WB
OCT4	proteintech	11263–1-AP	1:1000	WB
Nanog	CST	4903	1:2000	WB
SOX2	CST	3579	1:1000	WB
NHE7	Invitrogen	PA5-106748	1:1000/1:100	WB/IHC
PRSS1	abcam	ab200997	1:1000/1:2000	WB/IHC
c-Fos	CST	2250P	1:1000	WB
CD9	proteintech	20597–1-AP	1:1000	WB
CD63	proteintech	20597–1-AP	1:1000	WB
ERP27	abcam	ab181172	1:1000/1:200	WB/IHC
anti-rabbit IgG	Servicebio	G1213	1:200	IHC
anti-mouse IgG	CST	7076P2	1:1000	WB
anti-rabbit IgG	CST	7074P2	1:1000	WB
GRP78	Invitrogen	MA5-27686	1:2000	WB
XBP1s	CST	27901S	1:1000	WB
Chop	CST	2895S	1:1000	WB

### Quantitative reverse transcriptase-polymerase chain reaction (qRT-PCR)

mRNA expression analysis in EC cells was conducted through qRT-PCR. Following total RNA isolation with TRIzol reagent (Ambion, USA, 15596018), cDNA synthesis was performed using 1 μg RNA with the RevertAid cDNA Synthesis Kit (Thermo, K1622). PCR amplification was carried out using SYBR Green Pro Taq HS Premix (ACCURATE, AG11718) on a StepOnePlus instrument (Thermo). Post-amplification melting curve analysis ensured reaction specificity. Expression levels were normalized to β-actin and calculated via the 2^−ΔΔCt^ method, with primer sequences detailed in Table 3.

**Table 3:** The list of primers

Primer	Forward (5’−3’)	Reverse (5’−3’)
β-actin	CATGTACGTTGCTATCCAGGC	CTCCTTAATGTCACGCACGAT
CERS6	ACTGCTTCTGGTCTTACTTG	ATGTAAAGGGTTCCACTTCC
SATB1	AACAATGTGAGTGATCCGAA	AGCATGGTTCCTTTCCTAAG
EGR1	CCCCGACTACCTGTTTCCAC	TGGGTTTGATGAGCTGGGAC
PRSS1	AATTCTGGCTACCACTTC	GTCATTGTTCAGAGTCTTC
ERP27	CATTGATGCCACCAAATTGA	TAACCCAATCACAGTCACAG

### Enzyme-Linked Immunosorbent Assay (ELISA)

PRSS1 secretion was measured using a commercial ELISA kit (Yifeixue, YFXEH01825). Following centrifugation of culture supernatants (1000 × g, 20 min), standards and samples (50 μL/well) were processed according to the manufacture's instructions, including incubation with enzyme-conjugated antibody (37 °C, 30 min) and TMB substrate (10 min, dark). Reactions were stopped and absorbance was read at 450 nm, with concentrations determined from the standard curve.

### Exosomes isolation

Exosomes were purified from culture supernatant using differential centrifugation (Beckman Coulter Optima XPN-100). Sequential centrifugation steps included: 300 × g (10 min), 2,000 × g (10 min), and 10,000 × g (30 min) at 4 °C, followed by ultracentrifugation (120,000 × g, 70 min,4℃). The pellet was washed with PBS and re-centrifuged (120,000 × g, 70 min, 4℃), and resuspended in 100 μL PBS (−80 °C storage). WB was used to verified the expression of CD9 and CD63.

### Transmission Electron Microscopy (TEM) analysis of exosomes morphology

Exosome morphology was analyzed by TEM (Hitachi, HT7800). Samples (20 μL) were adsorbed onto carbon-coated grids (3–5 min, RT), excess sample was removed, followed by stained with 2% phosphotungstic acid (1–2 min), and air-dried. Digital images were captured to evaluate size, shape, and membrane integrity.

### Immunohistochemistry (IHC)

Protein expression of NHE7, PRSS1, and ERP27 was examined by IHC in tissue specimens. Following standard paraffin processing (embedding, 5–6 μm sectioning, deparaffinization), antigen retrieval was performed with citrate buffer (Servicebio, G1202). Endogenous peroxidase activity was quenched with 3% H₂O₂, and non-specific sites were blocked with 3% BSA (30 min). Primary antibody incubation (4 °C, overnight) was conducted followed by secondary antibody treatment (50 min, RT), DAB development, and hematoxylin counterstaining. This study involving human tissues was approved by the Ethics Committee of Women's Hospital, School of Medicine, Zhejiang University (Approval No. IRB-20250185-R). All procedures were performed in accordance with the ethical standards of the institutional research committee.

### Dual-luciferase reporter assay

The predicted c-Fos binding site within the PRSS1 promoter region and its mutated binding site (PRSS1-Mut) were amplified and cloned into the PGL3-basic vector (Promega). These reporter constructs (designated PRSS1-WT and PRSS1-Mut), along with the empty PGL3 control vector, were co-transfected into EC cells with pRL-TK using Lipofectamine 2000. Cells were lysed 24 h post-transfection, and firefly luciferase activity was measured using the Dual-Luciferase Reporter System (Promega) and normalized to Renilla luciferase activity. Triplicate biological replicates were performed.

### Chromatin immunoprecipitation (ChIP)

ChIP assay was conducted to verify c-Fos binding to the PRSS1 promoter. EC cells were crosslinked (1% formaldehyde, 37 °C, 10 min), lysed, and chromatin was sheared to 200–1000 bp. After centrifugation, 40 μL of the diluted lysate was reserved as the Input control. The remaining lysate was immunoprecipitated with anti-c-Fos antibodies (4 °C overnight) followed by Protein A/G bead capture. Complexes were washed, eluted, and crosslinks were reversed by incubation with 5 M NaCl at 65 °C for 4 h. DNA was purified and analyzed by qPCR targeting specific PRSS1 promoter regions, with enrichment calculated relative to the IgG control (using the ΔΔCt method, normalized to input DNA). Triplicates ensured reproducibility.

### Animal models

Female BALB/c nude mice (4–5 weeks old) were kept in SPF conditions with regulated temperature (22 ± 1℃), humidity (50 ± 10%), and 12-h light/dark cycles. The mice were randomly divided into three groups (*n* = 6 per group) and subcutaneously injected with Ishikawa cells stably overexpressing NHE7 or control vector. For the model group, a mixture (1:1) of 2 × 10^6^ NHE7-overexpressing Ishikawa cells and 2 × 10^6^ parental Ishikawa cells was injected, while the control group received 4 × 10^6^ parental Ishikawa cells alone. The mice were intraperitoneally injected with 4-PBA solution (150 mg/kg) for four consecutive days. Tumor growth was monitored every 2 days. When tumors reached the humane endpoint (tumor volume ≥ 1500 mm^3^) or showed signs of distress (e.g., > 20% weight loss, ulceration), mice were anesthetized with sodium pentobarbital (50 mg/kg, i.p.) and euthanized via cervical dislocation. Tumors were excised for final volume measurement and weighing. The animal experiments were approved by the Laboratory Animal Welfare and Ethics Committee of Women's Hospital, School of Medicine, Zhejiang University (Approval No. AE20250014). All procedures strictly followed the institutional guidelines for animal welfare and the ARRIVE guidelines.

### Statistical analysis

All statistical computations were conducted in GraphPad Prism version 6.0. For comparisons between two experimental groups, Student's t-test was employed, while one-way ANOVA was applied for analyses across multiple groups. Quantitative data are expressed throughout as mean ± standard deviation. Statistical significance was defined as *p* < 0.05 for all tests.

## Results

### NHE7 promotes the malignant phenotypes and activates the ER stress signaling cascades in EC cells

**Fig. 1:**
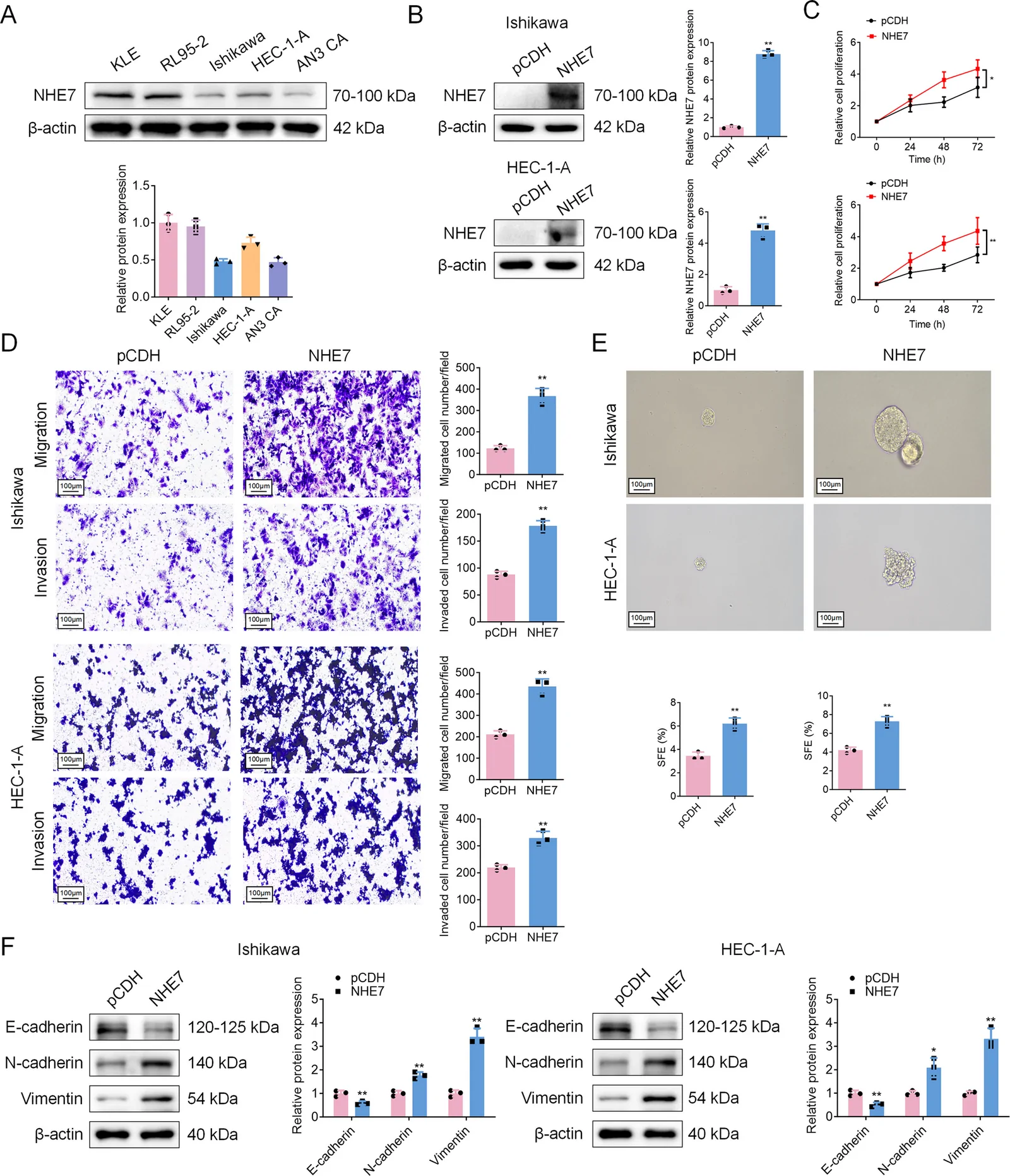
NHE7 promoted the malignant phenotypes in EC cells. **A** The endogenous expression of NHE7 in KLE, RL95-2, Ishikawa, HEC-1-A, and AN3 CA cells was detected by WB analysis. **B** NHE7 was overexpressed in Ishikawa and HEC-1-A cells, and the transfection efficiency was validated by WB analysis. **C** The MTT assay was performed to evaluate the proliferation of Ishikawa and HEC-1-A cells overexpressing NHE7. **D** Transwell assays were performed to assess cell migration and invasion capabilities. **E** Sphere formation assays were conducted to detect the tumor sphere-forming capacity. **F** The expression levels of EMT-related proteins were detected by WB assay, with β-actin serving as the loading control. All data were derived from at least three independent experiments. **p < 0.01, *p < 0.05 versus the control group

To determine the expression pattern of NHE7 in endometrial cancer, the endogenous protein levels of NHE7 were first examined across multiple EC cell lines using western blot analysis. The results showed that NHE7 expression was relatively low in Ishikawa and HEC-1-A cells, whereas it was markedly higher in KLE and RL95-2 cells (Fig. 1A). Based on this expression profile, Ishikawa and HEC-1-A cells were selected for gain-of-function studies to investigate the oncogenic roles of NHE7. NHE7 was overexpressed in Ishikawa and HEC-1-A cells, and the transfection efficiency was validated by WB analysis (Fig. 1B), confirming the successful establishment of NHE7-overexpressing models for subsequent functional assays. Functional assays demonstrated that NHE7 overexpression significantly enhanced cell proliferation, migration, and invasion (Fig. 1C&D). Furthermore, NHE7 overexpression augmented tumor sphere-forming efficiency, indicating enhanced self-renewal capacity and stemness properties in EC cells (Fig. 1E). Consistent with the pro-metastatic phenotypes, NHE7 overexpression induced epithelial-mesenchymal transition (EMT), evidenced by downregulation of the epithelial marker (E-cadherin) and upregulation of mesenchymal markers (N-cadherin and Vimentin) in EC cells (Fig. 1F). These findings establish NHE7 as a critical regulator promoting malignant phenotypes and stem-like characteristics in EC cells, underscoring its potential significance in EC progression.

**Fig. 2:**
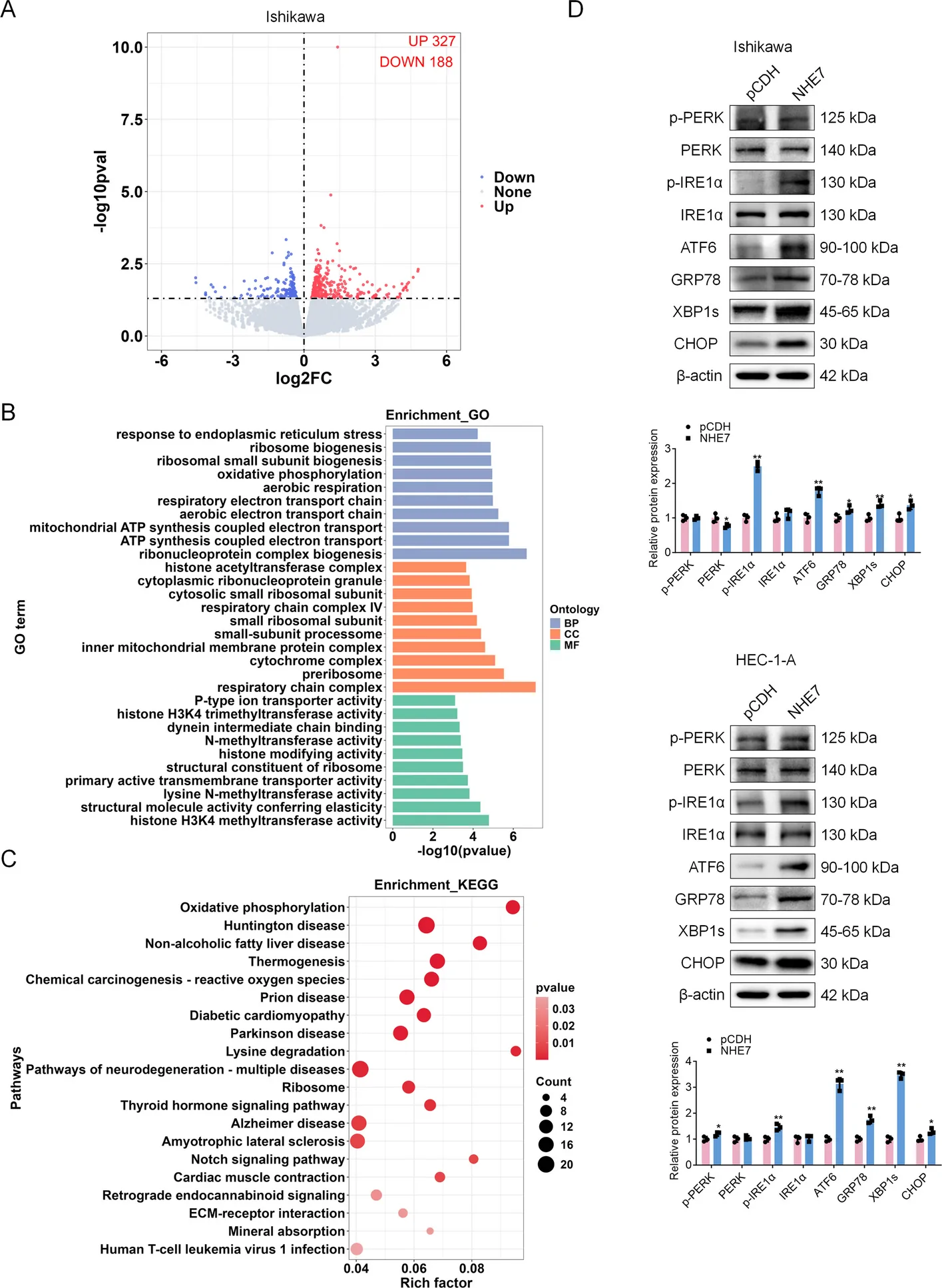
NHE7 activated the ER stress signaling cascades in EC cells. Transcriptome sequencing analysis was performed to identify differentially expressed genes (DEGs) between control and NHE7-overexpressing Ishikawa cells (*n* = 3). **A** DEGs between the two groups were analyzed, and their distribution was visualized using a volcano plot. **B **& **C** Functional enrichment analysis of DEGs was performed for (**B**) biological processes via GO analysis and (**C**) signaling pathways via KEGG analysis. **D** WB assay was used to detect the expression of ER stress-related proteins (p-PERK, PERK, p-IRE1α, IRE1α, ATF6, GRP78, XBP1s, and CHOP) in Ishikawa and HEC-1-A cells, with β-actin serving as the loading control. **p < 0.01, *p < 0.05 versus the control group

To elucidate the mechanisms underlying NHE7-mediated malignant phenotypes, transcriptomic analysis was performed using NHE7-overexpressing Ishikawa cells and corresponding vector controls. Differential expression analysis identified 327 upregulated and 188 downregulated genes (DEGs) upon NHE7 overexpression (Fig. 2A). GO and KEGG enrichment analyses revealed that these DEGs were predominantly enriched in the response to endoplasmic reticulum stress and oxidative phosphorylation (Fig. 2B&C). To validate the activation of ER stress signaling, WB assay was used to detect the expression of ER stress-related proteins, including phosphorylated protein kinase RNA-like endoplasmic reticulum kinase (p-PERK), PERK, phosphorylated inositol-requiring enzyme 1 alpha (p-IRE1α), IRE1α, activating transcription factor 6 (ATF6), glucose-regulated protein 78 (GRP78), X-box binding protein 1 spliced (XBP1s), and C/EBP homologous protein (CHOP) in Ishikawa and HEC-1-A cells. The results showed that NHE7 overexpression promoted the phosphorylation of PERK and IRE1α and increased the expression levels of ATF6, GRP78, XBP1s, and CHOP (Fig. 2D). These bioinformatic and experimental findings indicate that NHE7 activates the ER stress signaling cascades in EC cells.

**Fig. 3:**
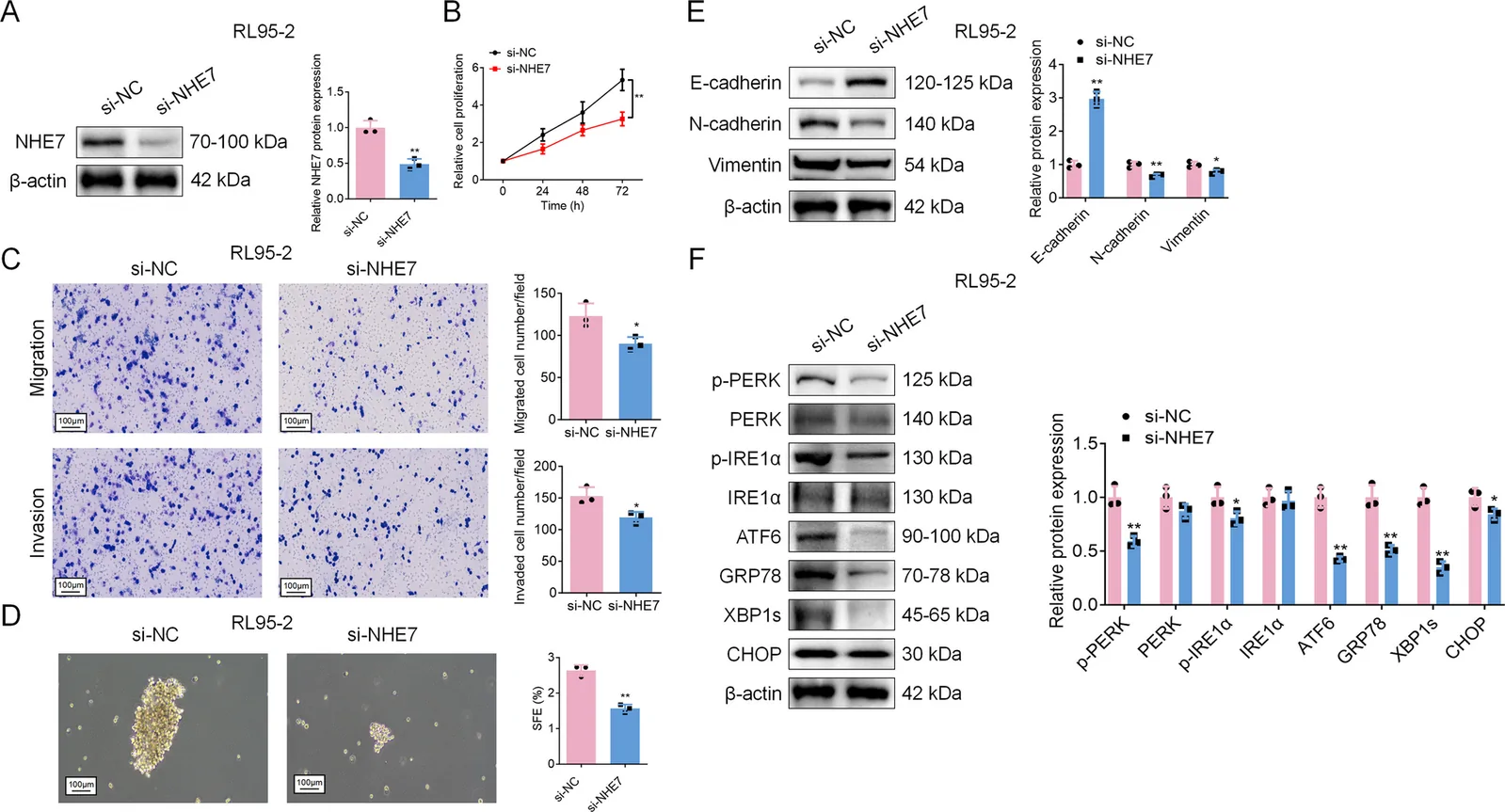
Knockdown of NHE7 suppressed the malignant phenotypes and inhibited ER stress signaling cascades in RL95-2 cells. **A** The knockdown efficiency of NHE7 in RL95-2 cells was validated by WB analysis. **B** The MTT assay was performed to evaluate the proliferation of RL95-2 cells after NHE7 knockdown. **C** Transwell assays were performed to assess cell migration and invasion capabilities. **D** Sphere formation assays were conducted to detect the tumor sphere-forming capacity. **E** The expression levels of EMT-related proteins were detected by WB assay, with β-actin serving as the loading control. **F** WB assay was used to detect the expression of ER stress-related proteins including p-PERK, PERK, p-IRE1α, IRE1α, ATF6, GRP78, XBP1s, and CHOP in RL95-2 cells, with β-actin serving as the loading control. All data were derived from at least three independent experiments. **p < 0.01, *p < 0.05 versus the control group

Loss-of-function experiments were performed in RL95-2 cells, which exhibited high endogenous NHE7 expression (as shown in Fig. 1A). The knockdown efficiency of NHE7 in RL95-2 cells was validated by WB analysis (Fig. 3A). The MTT assay demonstrated that NHE7 knockdown significantly suppressed cell proliferation (Fig. 3B). Transwell assays showed that NHE7 knockdown reduced cell migration and invasion capabilities (Fig. 3C). Sphere formation assays further revealed that NHE7 knockdown impaired tumor sphere-forming capacity (Fig. 3D). Moreover, WB analysis showed that NHE7 knockdown reversed EMT progression, as evidenced by increased E-cadherin expression and decreased N-cadherin and Vimentin expression (Fig. 3E). Finally, WB assay was used to detect the expression of ER stress-related proteins and the results showed that NHE7 knockdown suppressed the activation of ER stress signaling cascades (Fig. 3F). Collectively, these results indicate that NHE7 promotes malignant phenotypes and activates ER stress signaling cascades in EC cells.

### NHE7 enhances the malignant phenotypes of recipient EC cells through ER stress-dependent intercellular signaling transmission

**Fig. 4:**
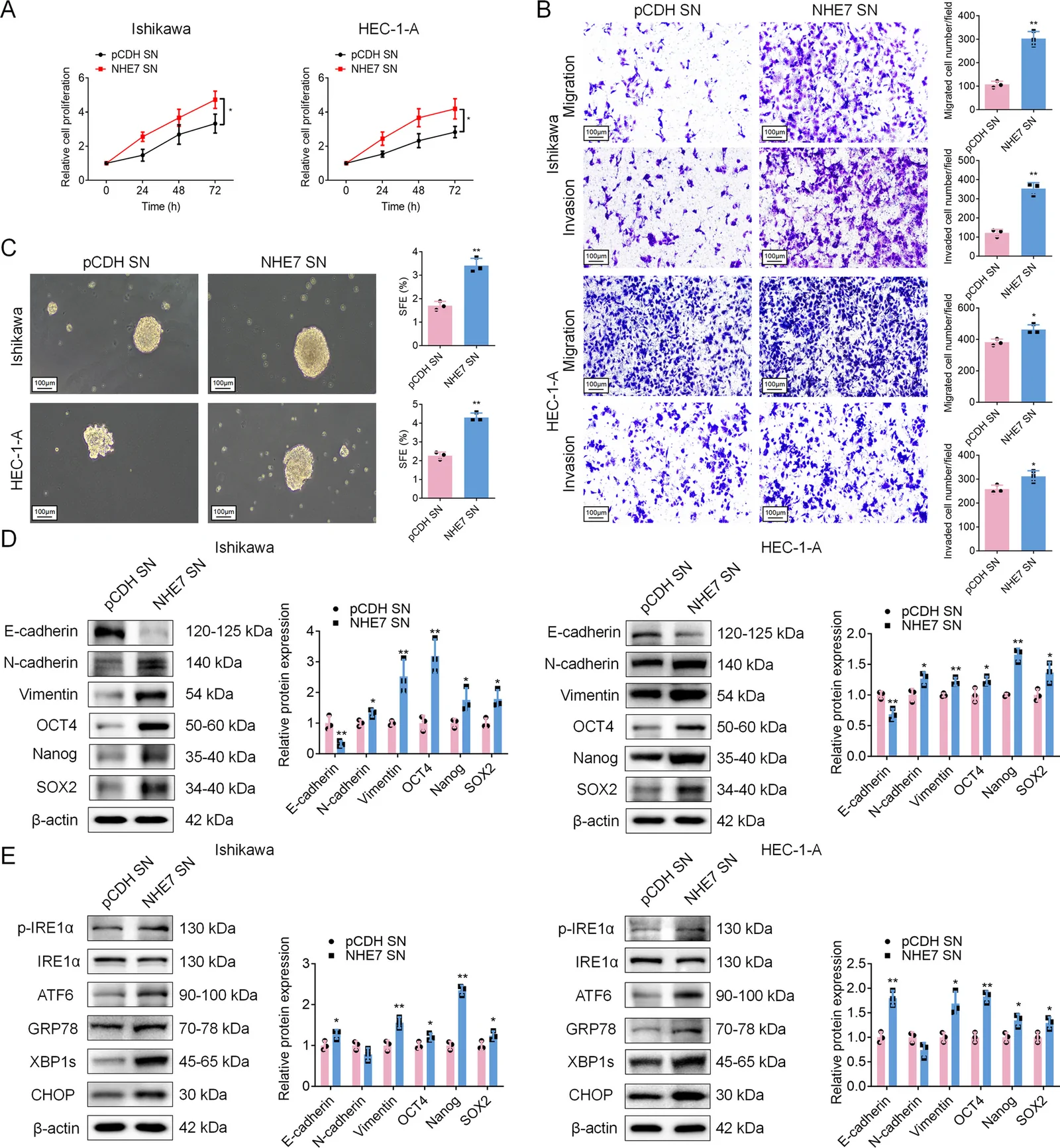
Treatment with supernatants from NHE7-overexpressing Ishikawa cells effectively induced malignant phenotypes and ER stress signaling in recipient EC cells. Ishikawa and HEC-1-A cells were incubated with supernatants from NHE7-overexpressing Ishikawa cells. **A** The MTT assay was performed to evaluate the proliferation of Ishikawa and HEC-1-A cells. **B** Transwell assays were performed to assess the migration and invasion capabilities. **C** Sphere formation assays were conducted to detect the tumor sphere-forming capacity. **D** The expression levels of EMT-related proteins and stemness-related proteins were detected by WB assay. **E** WB assay was used to detect the expression of ER stress-related proteins (p-IRE1α, IRE1α, ATF6, GRP78, XBP1s, and CHOP). β-actin served as the loading control. All data were derived from at least three independent experiments. **p < 0.01, *p < 0.05 versus the control group

Given the established role of ER stress signaling in tumorigenesis and progression, whether NHE7 influences the malignant phenotypes of EC cells through intercellular transmission of ER stress-related signals was further investigated. To test this hypothesis, conditioned supernatant (SN) was collected from NHE7-overexpressing EC cells (donor cells) after 48 h of culture. This SN contains all secreted factors released by donor cells, including exosomes, soluble proteins, and other bioactive molecules. This SN was then applied to naïve recipient EC cells to determine whether NHE7-driven secreted factors are sufficient to alter recipient cell behavior. As shown in Fig. 4, treatment with NHE7 SN significantly enhanced recipient EC cell proliferation, migration, and invasion compared with control SN (Fig. 4A&B). Furthermore, exposure to NHE7-overexpressing SN promoted tumor sphere formation and upregulated the expression of stemness-related markers, including OCT4 (Octamer-binding Transcription Factor 4), Nanog (NANOG Homeobox Protein), and SOX2 (SRY-box Transcription Factor 2) (Fig. 4C&D). NHE7 SN also promoted the EMT process in treated recipient EC cells (Fig. 4D). Importantly, treatment with NHE7 SN effectively induced ER stress signaling in recipient EC cells (Fig. 4E).

**Fig. 5:**
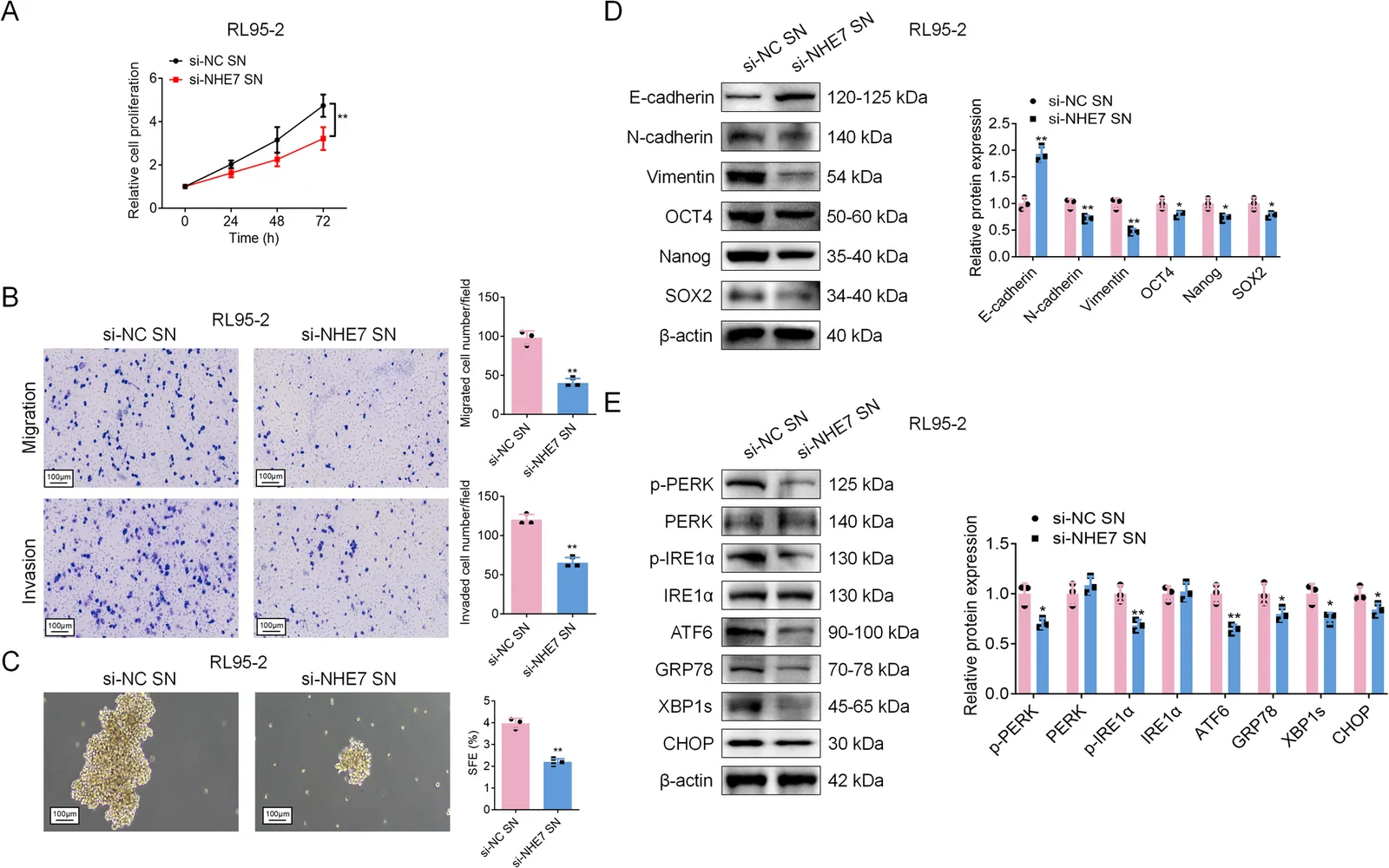
Knockdown of NHE7 in RL95-2 cells suppressed malignant phenotypes and ER stress signaling in recipient EC cells. RL95-2 cells were incubated with supernatants from NHE7-knockdown RL95-2 cells. **A** The MTT assay was performed to evaluate the proliferation of RL95-2 cells. **B** Transwell assays were performed to assess the migration and invasion capabilities. **C** Sphere formation assays were conducted to detect the tumor sphere-forming capacity. **D** The expression levels of EMT-related proteins and stemness-related proteins were detected by WB assay. **E** WB assay was used to detect the expression of ER stress-related proteins (p-PERK, PERK, p-IRE1α, IRE1α, ATF6, GRP78, XBP1s, and CHOP). β-actin served as the loading control. All data were derived from at least three independent experiments. **p < 0.01, *p < 0.05 versus the control group

Conversely, to further validate the role of NHE7 in intercellular signaling transmission, loss-of-function experiments were performed using SN from NHE7-knockdown RL95-2 cells. As shown in Fig. 5, treatment with SN from NHE7-knockdown RL95-2 cells significantly suppressed recipient RL95-2 cell proliferation, migration, and invasion (Fig. 5A&B). Sphere formation capacity was also impaired, and the expression of EMT-related proteins and stemness-related markers was reduced (Fig. 5C&D). Moreover, NHE7-knockdown SN effectively inhibited ER stress signaling in recipient RL95-2 cells (Fig. 5E). These results further confirm that NHE7-mediated secreted factors play a crucial role in transmitting malignant phenotypes and ER stress signals to recipient EC cells.

**Fig. 6:**
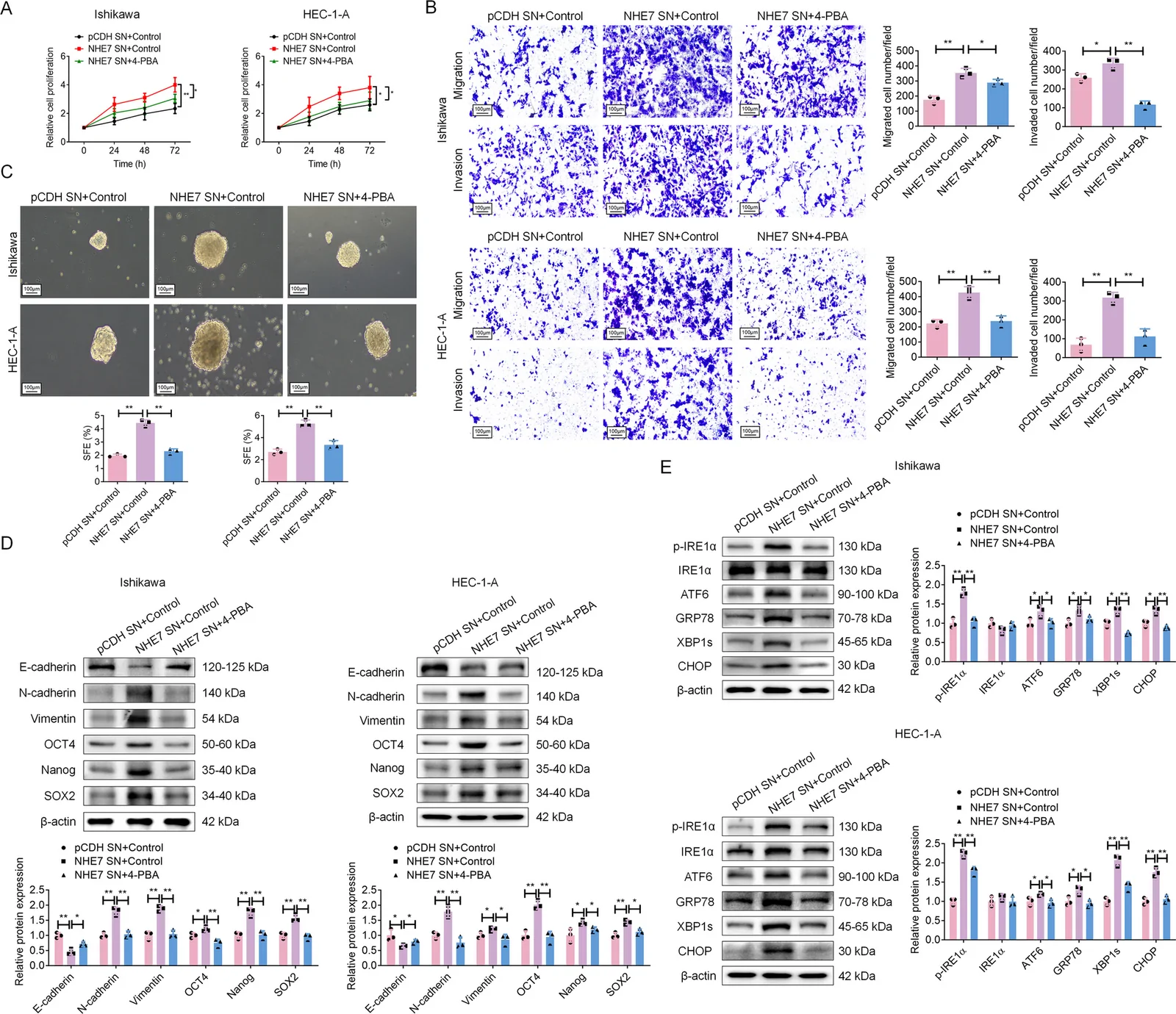
NHE7 enhanced the malignant phenotypes of recipient EC cells through ER stress-dependent intercellular signaling transmission. Ishikawa and HEC-1-A cells were incubated with supernatants from NHE7-overexpressing Ishikawa cells, and the ER stress inhibitor 4-PBA solution(10 μM) was added to the culture for 24 h. **A** The MTT assay was performed to evaluate the proliferation of Ishikawa and HEC-1-A cells. **B** Transwell assays were performed to assess the migration and invasion capabilities of Ishikawa and HEC-1-A cells. **C** Sphere formation assays were conducted to detect the tumor sphere-forming capacity. **D** The expression levels of EMT-related proteins and stemness-related proteins were detected by WB assay. **E** WB assay was used to detect the expression of ER stress-related proteins (p-IRE1α, IRE1α, ATF6, GRP78, XBP1s, and CHOP). β-actin served as the loading control. All data were derived from at least three independent experiments. **p < 0.01, *p < 0.05 versus the control group

To confirm the role of ER stress in mediating these effects, the ER stress inhibitor 4-phenylbutyric acid (4-PBA) was administered to treat recipient EC cells. Notably, 4-PBA treatment significantly attenuated the NHE7 SN-mediated promotion of proliferation, migration, invasion, stemness maintenance, and EMT process (Fig. 6A-D). Consistently, 4-PBA treatment suppressed the NHE7 SN-induced increase in the expression of ER stress-related proteins (Fig. 6E). These results suggest that NHE7 enhances the malignant phenotypes and stemness of recipient EC cells through ER stress-dependent intercellular signaling transmission.

### NHE7 promotes exosome-mediated ER stress signaling and malignant phenotypes through upregulation of PRSS1 in EC cells

**Fig. 7:**
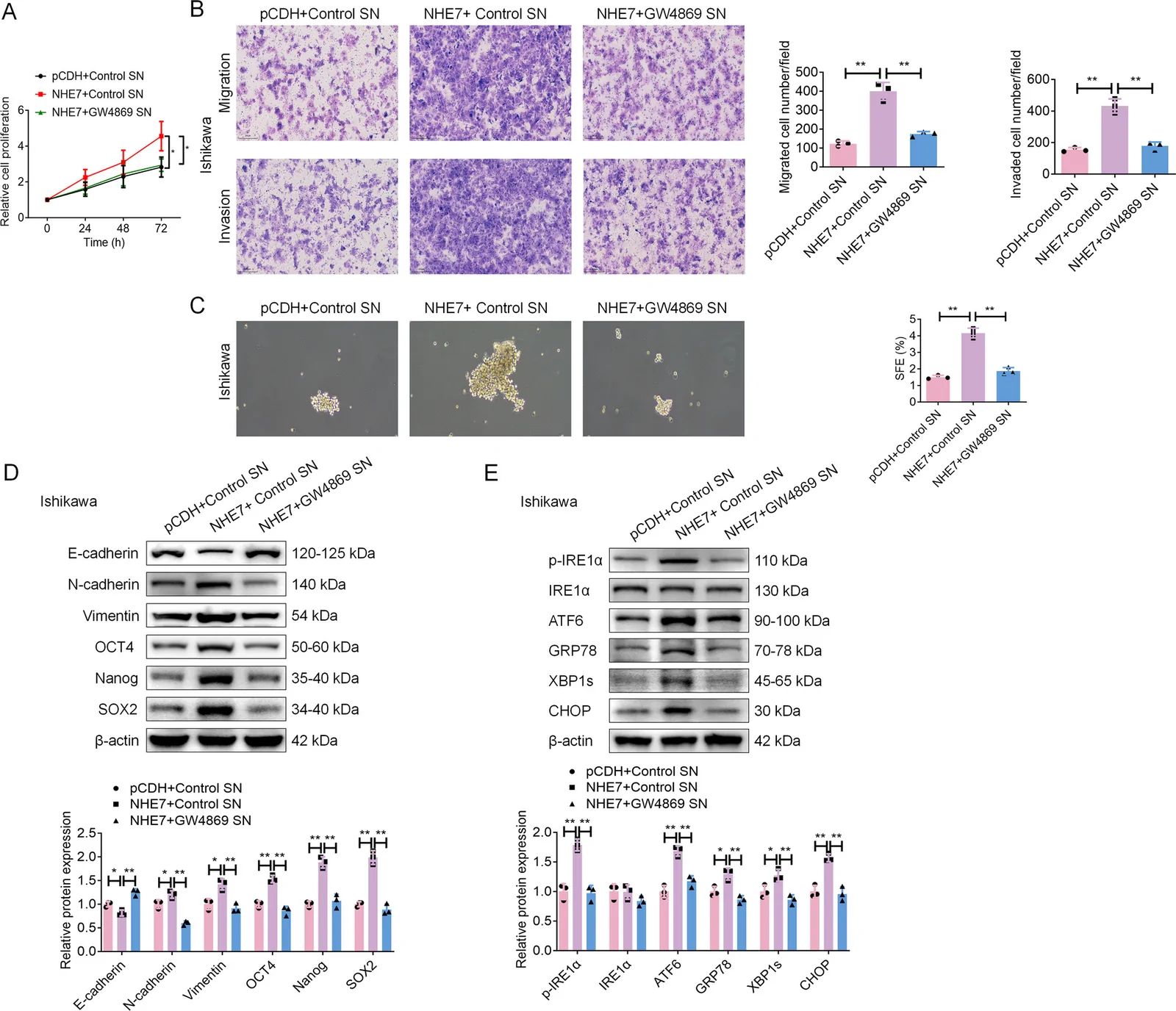
NHE7 enhanced the malignant phenotypes and ER stress signaling of recipient EC cells through exosome-mediated intercellular signaling transmission. Donor Ishikawa cells overexpressing NHE7 were pretreated with the exosome inhibitor GW4869 (10 μM) for 24 h. The resulting supernatants were then collected and used to incubate recipient Ishikawa cells. **A** The MTT assay was performed to evaluate the proliferation of Ishikawa cells. **B** Transwell assays were performed to assess the migration and invasion capabilities of Ishikawa cells. **C** Sphere formation assays were conducted to detect the tumor sphere-forming capacity. **D** The expression levels of EMT-related proteins and stemness-related proteins were detected by WB assay. **E** WB assay was used to detect the expression of ER stress-related proteins (p-IRE1α, IRE1α, ATF6, GRP78, XBP1s, and CHOP). β-actin served as the loading control. All data were derived from at least three independent experiments. **p < 0.01, *p < 0.05 versus the control group

To investigate whether NHE7 transmits malignant signals through exosome-mediated intercellular communication, the exosome inhibitor GW4869 (10 μM) was added to donor NHE7-overexpressing Ishikawa cells for 24 h. The results showed that GW4869 treatment significantly attenuated NHE7 supernatant-induced cell proliferation, migration, and invasion (Fig. 7A&B). Furthermore, GW4869 treatment suppressed tumor sphere formation and reduced the expression of EMT-related proteins and stemness-related markers induced by NHE7-overexpressing supernatant (Fig. 7C&D). Consistently, GW4869 treatment inhibited the activation of ER stress signaling (Fig. 7E). These findings indicate that NHE7 enhances malignant phenotypes and ER stress signaling in recipient EC cells through exosome-mediated intercellular transmission.

**Fig. 8:**
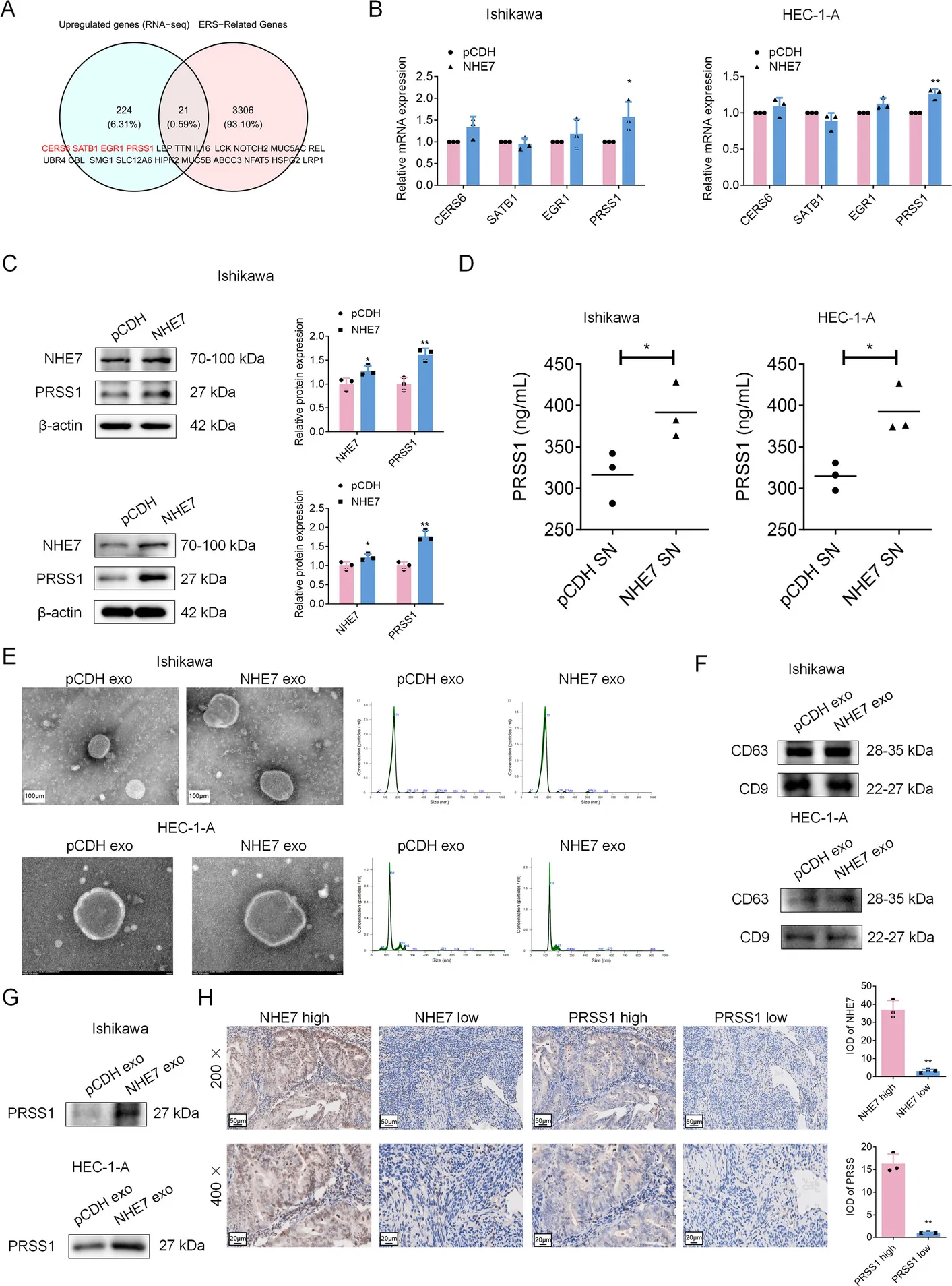
PRSS1 was identified as a target of NHE7 and showed a positive correlation with NHE7 expression. **A** Venn diagram showing the overlap between DEGs in NHE7-overexpressing Ishikawa cells versus control groups and the canonical ER stress gene set. The intersecting region represents NHE7-regulated ER stress-related genes. **B** The mRNA expression levels of NHE7-regulated ER stress-related genes in Ishikawa and HEC-1-A cells were determined using qRT-PCR. **C** WB assay was performed to detect the protein expression levels of PRSS1 and NHE7 in Ishikawa and HEC-1-A cells overexpressing NHE7. **D** The expression level of PRSS1 in the supernatants of Ishikawa and HEC-1-A cells overexpressing NHE7 was detected by ELISA. **E** TEM images of exosomes isolated from Ishikawa and HEC-1-A cells were shown. **F** WB assay was performed to detect the expression levels of CD9 and CD63 in exosomes. **G** WB assay was performed to detect the expression level of PRSS1 in exosomes. **H** IHC assay was used to detect the expression levels of NHE7 and PRSS1 in EC tissues. All data were derived from at least three independent experiments. **p < 0.01, *p < 0.05 versus the control group

To investigate the underlying mechanism of NHE7 in EC cells, the identified NHE7-associated DEGs (Fig. 2A) were intersected with an ER stress-related gene set from the GeneCards database. This overlap was visualized using a Venn diagram (Fig. 8A). Among the 21 intersecting genes, further survival analysis using TCGA endometrial tumor samples identified nine genes significantly associated with poor prognosis (Figure S1A), including CERS6 (Ceramide Synthase 6), SATB1 (Special AT-Rich Sequence Binding Protein 1), EGR1 (Early Growth Response 1), and PRSS1 (Serine Protease 1). Based on their documented functional relevance to cancer progression [18–21], these four genes were selected for further investigation. The qRT-PCR analysis in NHE7-overexpressing Ishikawa and HEC-1-A cells revealed that PRSS1 exhibited the most significant upregulation among these genes upon NHE7 overexpression (Fig. 8B). PRSS1 encodes trypsinogen 1, a serine protease that catalyzes the hydrolysis of peptide substrates and has been implicated in digestion, inflammation, and tumorigenesis. Aberrant activation or overexpression of PRSS1 is reported in various pathological conditions [21]. Based on its pronounced induction and biological potential, PRSS1 was prioritized as a candidate downstream target of NHE7 for further investigation.

Consistent with transcriptional data, NHE7 overexpression significantly increased PRSS1 protein expression in Ishikawa and HEC-1-A cells (Fig. 8C) and elevated PRSS1 levels in the supernatant (Fig. 8D). Exosomes (NHE7 exo) isolated from NHE7-overexpressing Ishikawa and HEC-1-A cells were characterized via electron microscopy and exosomal marker (CD63 and CD9) detection (Fig. 8E&F). Notably, PRSS1 expression was significantly upregulated in NHE7-overexpressing exosomes (NHE7 exo) compared to controls (Fig. 8G), indicating that PRSS1 may function as a secretory protein capable of modulating recipient EC cells. Furthermore, to validate the correlation between NHE7 and PRSS1 expression in vivo, IHC analysis was performed on serial sections from clinical EC samples. The results revealed that tissues with high NHE7 expression exhibited correspondingly elevated PRSS1 levels in the adjacent sections, suggesting a positive correlation between NHE7 and PRSS1 expression in vivo (Fig. 8H).

**Fig. 9:**
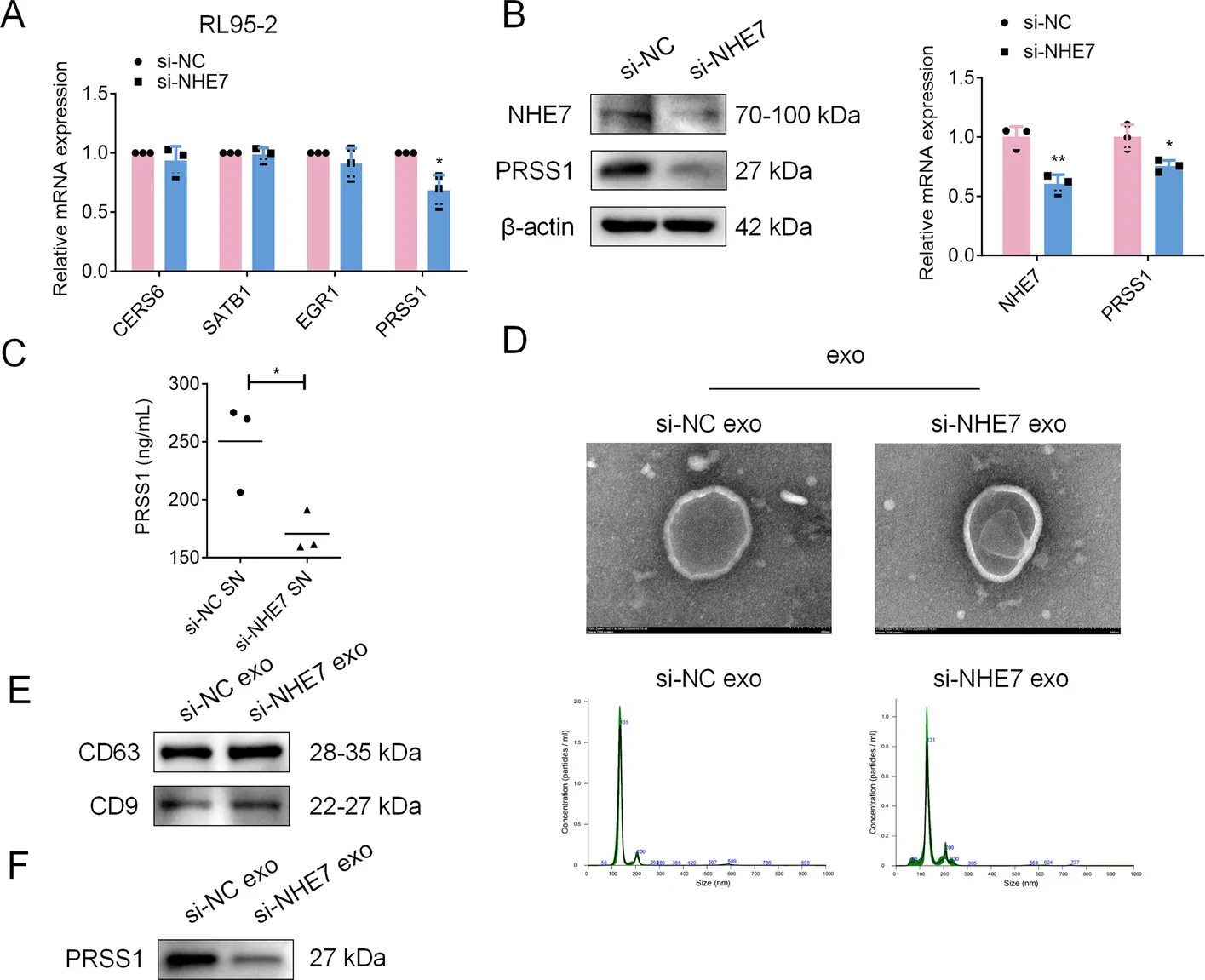
PRSS1 was identified as a downstream target of NHE7 in RL95-2 cells. **A** The mRNA expression levels of NHE7-regulated ER stress-related genes in RL95-2 cells were determined using qRT-PCR. **B** WB assay was performed to detect the protein expression levels of PRSS1 and NHE7 in RL95-2 cells with NHE7 knockdown. **C** The expression level of PRSS1 in the supernatants of RL95-2 cells with NHE7 knockdown was detected by ELISA. **D** TEM images of exosomes isolated from RL95-2 cells with NHE7 knockdown were shown. **E** WB assay was performed to detect the expression levels of CD9 and CD63 in exosomes. **F** WB assay was performed to detect the expression level of PRSS1 in exosomes. All data were derived from at least three independent experiments. **p < 0.01, *p < 0.05 versus the control group

Loss-of-function experiments were performed in RL95-2 cells to further confirm that PRSS1 serves as a downstream target of NHE7 and mediates NHE7-regulated malignant phenotypes. The qRT-PCR analysis of NHE7-regulated ER stress-related genes in RL95-2 cells with NHE7 knockdown confirmed that PRSS1 expression was significantly reduced upon NHE7 silencing (Fig. 9A). WB analysis further demonstrated that NHE7 knockdown decreased both NHE7 and PRSS1 protein expression levels in RL95-2 cells (Fig. 9B). Consistently, ELISA results showed that PRSS1 levels in the supernatant of NHE7-knockdown RL95-2 cells were markedly decreased compared to controls (Fig. 9C). Exosomes isolated from NHE7-knockdown RL95-2 cells were characterized by TEM and exosomal markers CD9 and CD63 (Fig. 9D, 9E). Importantly, PRSS1 expression in exosomes derived from NHE7-knockdown RL95-2 cells was significantly reduced (Fig. 9F). These results collectively indicate that PRSS1 is a downstream target of NHE7 and mediates NHE7-regulated malignant phenotypes in EC cells.

Additionally, based on the median expression value of PRSS1, EC tumor samples from the TCGA database were divided into two groups: high- and low-PRSS1 expression. Differential expression analysis identified 901 upregulated DEGs and 329 downregulated DEGs in the PRSS1-high cohort (Figure S2A). Kaplan–Meier survival analysis demonstrated that patients with low PRSS1 expression had significantly better overall survival compared to those with high PRSS1 expression (Figure S2B), suggesting that elevated PRSS1 is associated with poor prognosis in EC. Consistently, PRSS1 expression was significantly higher in tumor tissues compared to adjacent non-tumor (Health) controls (Figure S2C). Notably, correlation analysis revealed a statistically significant weak positive correlation between PRSS1 and SLC9A7 (NHE7) expression (Figure S2D), further supporting their potential functional association in EC progression. Consistent with these findings, high NHE7 expression has previously been reported to be significantly associated with poor overall survival in EC patients [7].

### PRSS1 mediates NHE7-enhanced ER stress signaling and malignant phenotypes in recipient EC cells via exosomal transmission

**Fig. 10:**
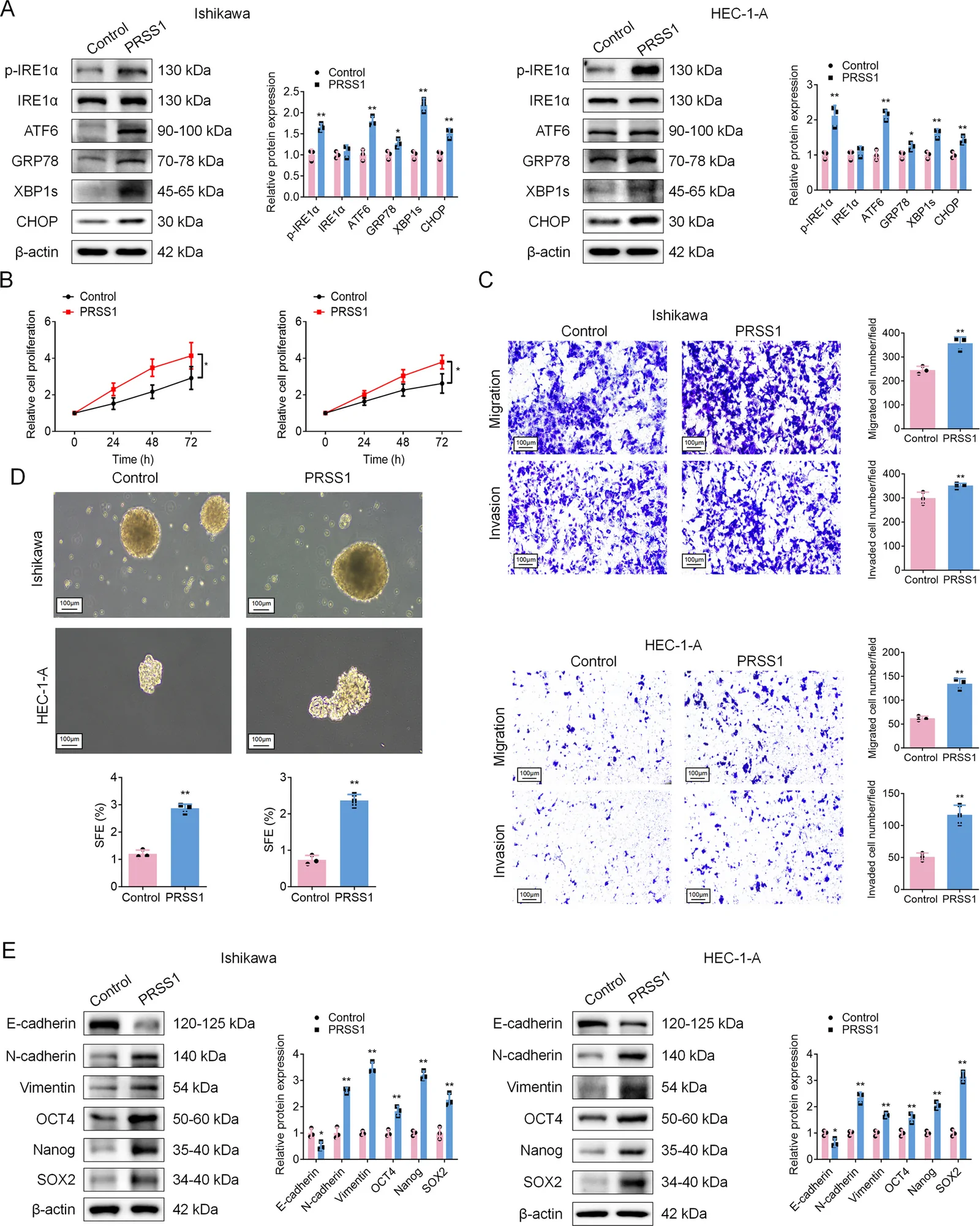
PRSS1 facilitated the malignant phenotypes and activated the ER stress signaling cascades in EC cells. Ishikawa and HEC-1-A cells were incubated with recombinant PRSS1 protein (100 ng/mL) for 24 h. **A** WB assay was used to detect the expression of ER stress-related proteins (p-IRE1α, IRE1α, ATF6, GRP78, XBP1s, and CHOP). **B** The MTT assay was performed to evaluate the proliferation of Ishikawa and HEC-1-A cells. **C** Transwell assays were performed to assess the migration and invasion capabilities of Ishikawa and HEC-1-A cells. **D** Sphere formation assays were conducted to detect the tumor sphere-forming capacity of EC cells. **E** The expression levels of EMT-related proteins and stemness-related proteins were detected by WB assay. β-actin served as the loading control. All data were derived from at least three independent experiments. **p < 0.01, *p < 0.05 versus the control group

To elucidate the role of PRSS1 in ER stress activation and malignant phenotypes of recipient EC cells, Ishikawa and HEC-1-A cells were treated with recombinant PRSS1 protein. The results demonstrated that treatment with PRSS1 led to increased expression levels of p-IRE1α, IRE1α, ATF6, GRP78, XBP1s, and CHOP, indicating activation of ER stress response (Fig. 10A). Functionally, PRSS1-treated cells exhibited augmented proliferation, migration, invasion, and tumor sphere formation (Fig. 10B-D). Consistently, PRSS1 facilitated the EMT process and enhanced cellular stemness in recipient EC cells (Fig. 10E).

**Fig. 11:**
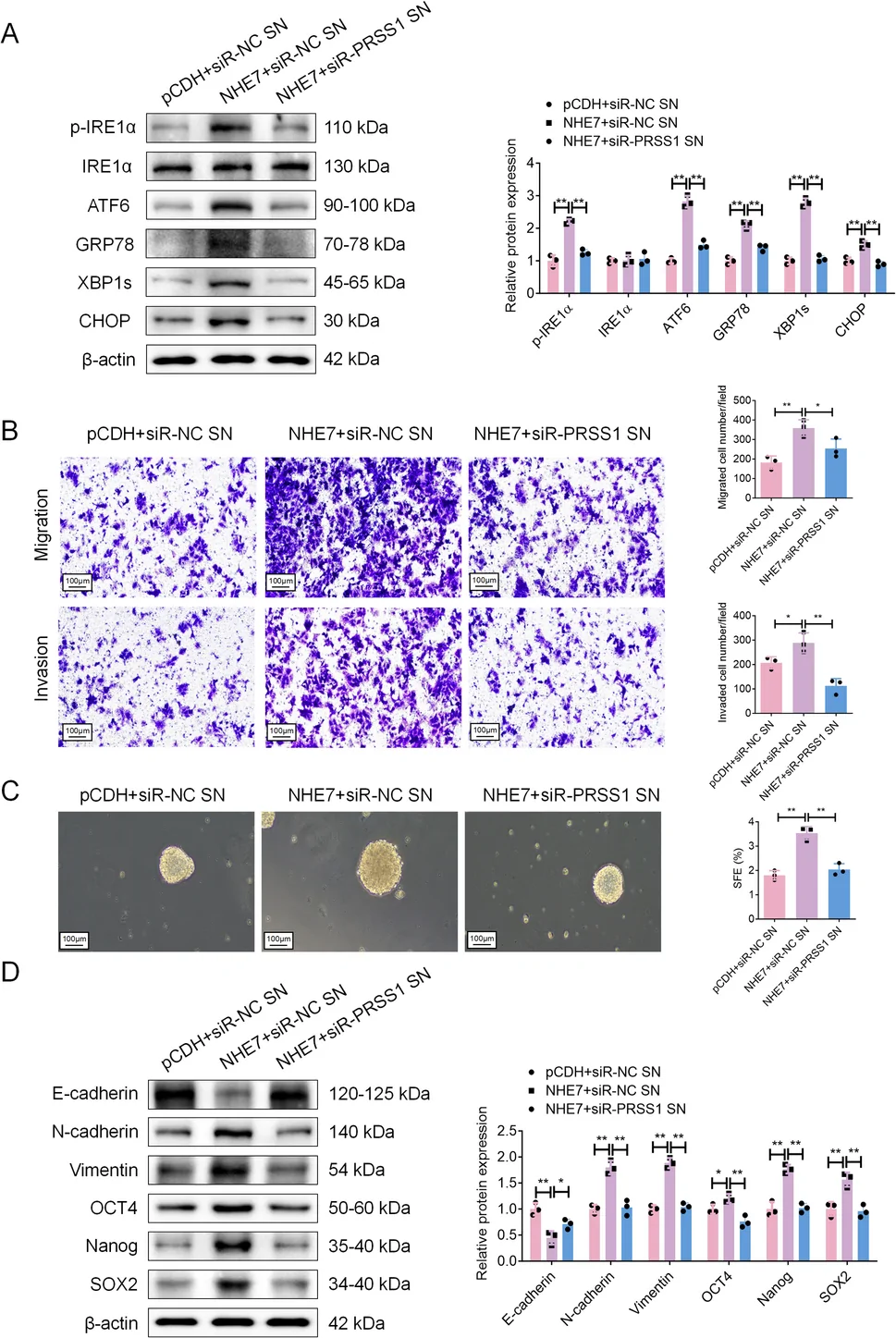
NHE7 enhanced ER stress signaling transmission and malignant phenotypes of EC cells by upregulating PRSS1 expression. Ishikawa cells were incubated with supernatants from NHE7 overexpression alone or in combination with PRSS1-knocked-down Ishikawa cells. **A** WB assay was used to detect the expression of ER stress-related proteins (p-IRE1α, IRE1α, ATF6, GRP78, XBP1s, and CHOP). **B** Transwell assays were performed to assess the migration and invasion capabilities of Ishikawa and HEC-1-A cells. **C** Sphere formation assays were conducted to detect the tumor sphere-forming capacity. **D** The expression levels of EMT-related proteins and stemness-related proteins were detected by WB assay. β-actin served as the loading control. All data were derived from at least three independent experiments. **p < 0.01, *p < 0.05 versus the control group

To ascertain whether NHE7 drives ER stress signaling transmission and cell malignant phenotypes through PRSS1 regulation, PRSS1 was knocked down in EC cells using small interfering RNAs (siRNAs). Among three siRNA candidates, siR-PRSS1-1 (48 h) showed the most efficient knockdown and was selected for subsequent experiments (Figure S3A). The supernatant from donor EC cells with NHE7 overexpression alone (NHE7 SN) or in combination with PRSS1 knockdown (NHE7 + siR-PRSS1 SN) was used to treat the corresponding recipient EC cells, and the ER stress response and phenotypic alterations of these treated cells were analyzed. The results revealed that treatment with NHE7 SN significantly enhanced the expression of ER stress markers in recipient EC cells, an effect that was attenuated by NHE7 + siR-PRSS1 SN (Fig. 11A). Furthermore, NHE7 SN promoted migratory and invasive capacities, stemness features, and EMT process. However, these NHE7 SN-induced malignant phenotypes and stemness were diminished by PRSS1 knockdown, at least partially (Fig. 11B-D). Collectively, these findings demonstrate that NHE7 enhances ER stress signaling transmission by promoting PRSS1 expression and secretion, which contributes to promoting the malignant phenotypes and stem-like characteristics of recipient EC cells.

**Fig. 12:**
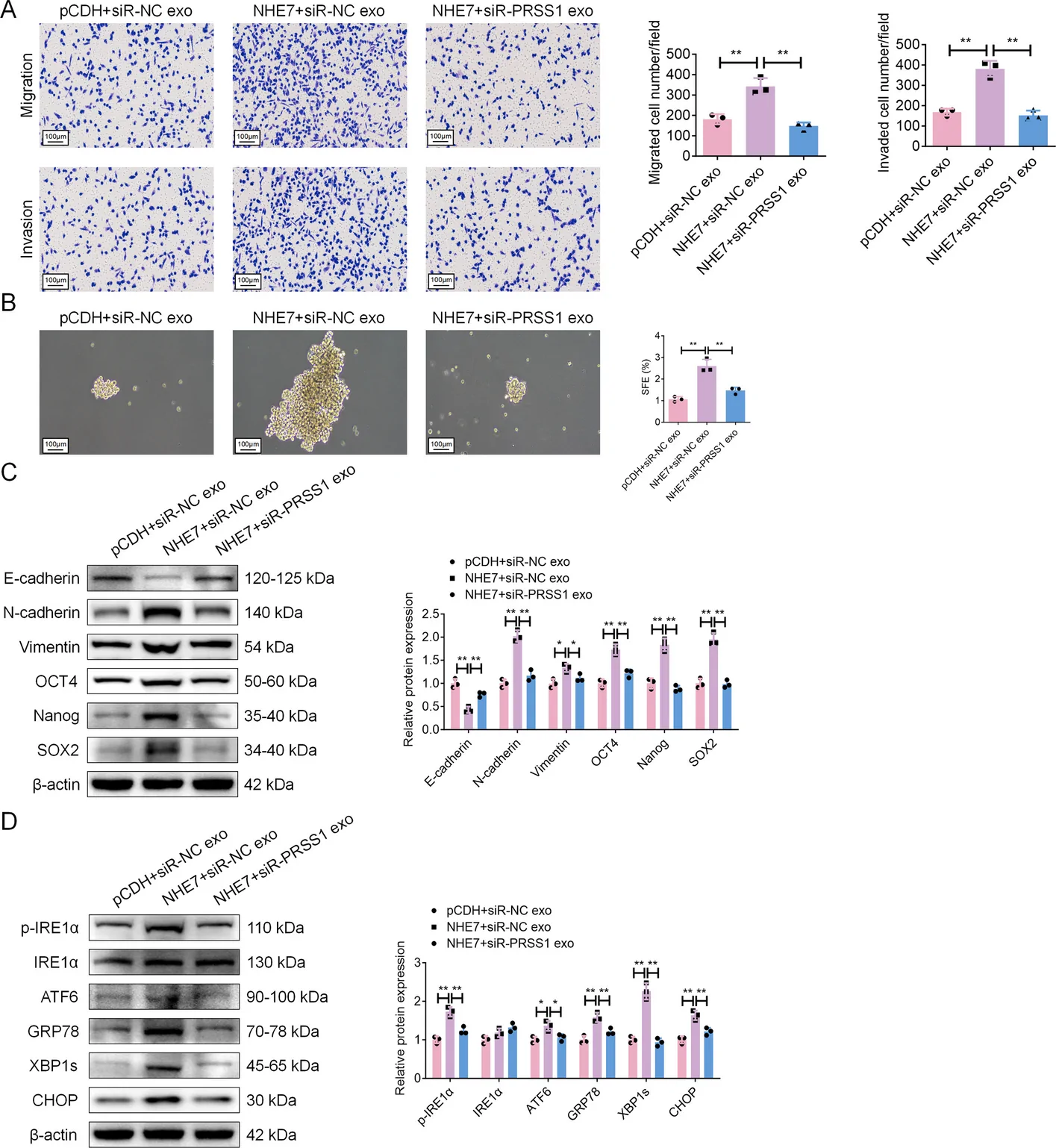
NHE7-enhanced ER stress signaling transmission and malignant phenotypes of EC cells were mediated by PRSS1-containing exosomes. Ishikawa cells were incubated with exosomes derived from NHE7-overexpressing Ishikawa cells alone or in combination with PRSS1-knocked-down Ishikawa cells. **A** Transwell assays were performed to assess the migration and invasion capabilities of Ishikawa cells. **B** Sphere formation assays were conducted to detect the tumor sphere-forming capacity. **C** The expression levels of EMT-related proteins and stemness-related proteins were detected by WB assay. **D** WB assay was used to detect the expression of ER stress-related proteins (p-IRE1α, IRE1α, ATF6, GRP78, XBP1s, and CHOP). β-actin served as the loading control. All data were derived from at least three independent experiments. **p < 0.01, *p < 0.05 versus the control group

Given that PRSS1 was identified as a cargo of NHE7-overexpressing exosomes (Fig. 8G), whether PRSS1-containing exosomes mediate NHE7-enhanced ER stress signaling and malignant phenotypes was further investigated. Ishikawa cells were incubated with exosomes derived from NHE7-overexpressing Ishikawa cells alone (NHE7 exo) or in combination with PRSS1-knocked-down Ishikawa cells (NHE7 + siR-PRSS1 exo) (Fig. 12). Functionally, NHE7 exo treatment enhanced the migration and invasion capacities of recipient Ishikawa cells (Fig. 12A), promoted tumor sphere formation (Fig. 12B), and increased the expression of EMT-related proteins and stemness-related markers (Fig. 12C). Notably, co-treatment with NHE7 + siR-PRSS1 exo partially reversed these malignant phenotypes, indicating that PRSS1-containing exosomes are critical mediators of NHE7-enhanced intercellular signaling transmission (Fig. 12A-C). Furthermore, NHE7 exo treatment significantly activated ER stress signaling in recipient Ishikawa cells, as evidenced by increased expression of p-IRE1α, IRE1α, ATF6, GRP78, XBP1s, and CHOP, whereas this effect was markedly reduced by PRSS1 knockdown (Fig. 12D).

**Fig. 13:**
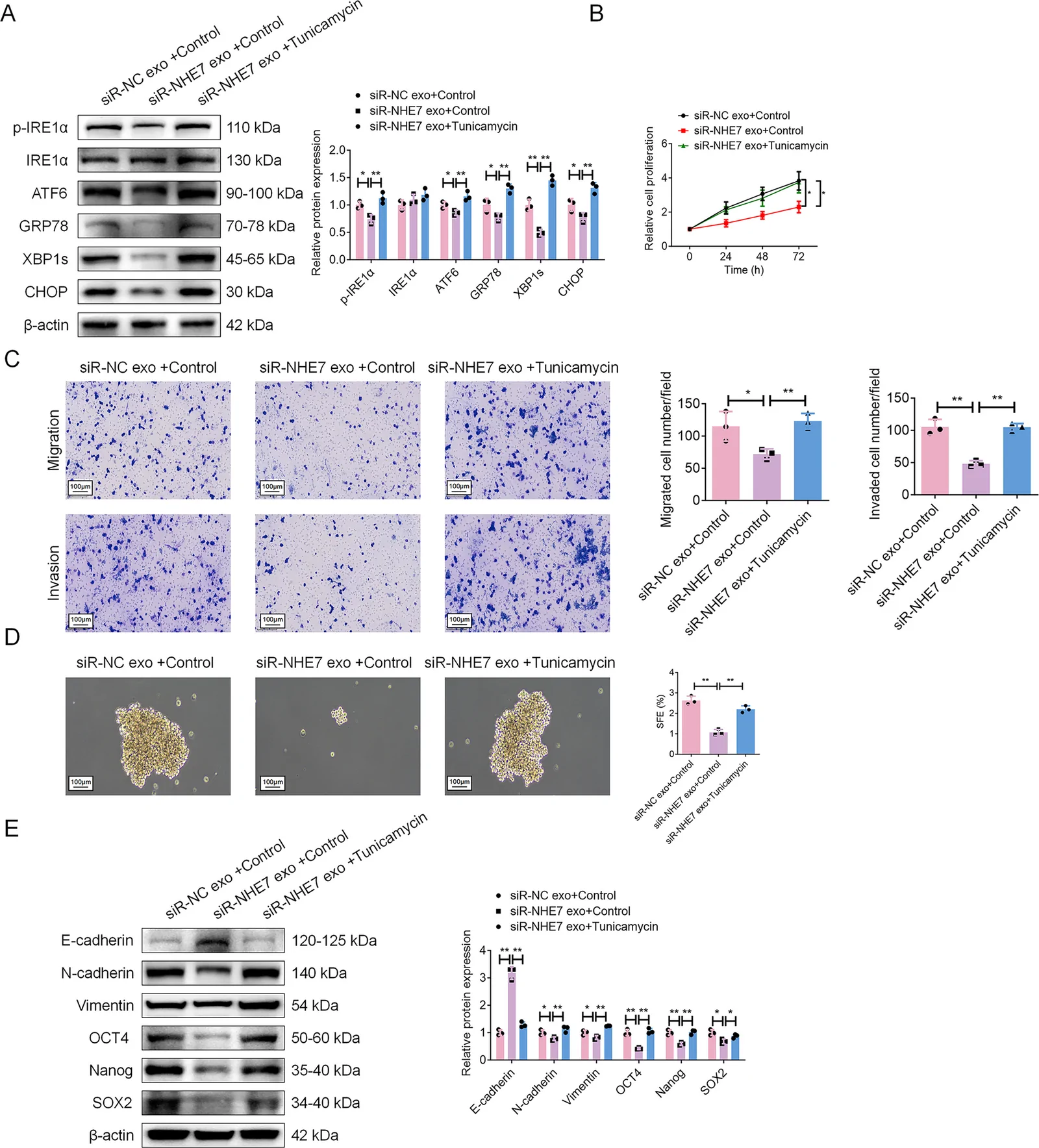
NHE7 knockdown suppressed the malignant phenotypes and stemness of recipient RL95-2 cells through exosome-mediated intercellular signaling transmission, which was rescued by ER stress activation. RL95-2 cells were incubated with exosomes derived from NHE7-knockdown RL95-2 cells, with or without the ER stress inducer Tunicamycin treatment (10 μg/mL) for 24 h. **A** WB assay was used to detect the expression of ER stress-related proteins (p-IRE1α, IRE1α, ATF6, GRP78, XBP1s, and CHOP). **B** The MTT assay was performed to evaluate the proliferation of RL95-2 cells. **C** Transwell assays were performed to assess the migration and invasion capabilities of RL95-2 cells. **D** Sphere formation assays were conducted to detect the tumor sphere-forming capacity. **E** The expression levels of EMT-related proteins and stemness-related proteins were detected by WB assay. β-actin served as the loading control. All data were derived from at least three independent experiments. **p < 0.01, *p < 0.05 versus the control group

Whether NHE7 knockdown suppresses malignant phenotypes through exosome-mediated signaling and whether this effect can be rescued by ER stress activation were further investigated. RL95-2 cells were incubated with exosomes derived from NHE7-knockdown RL95-2 cells, with or without the ER stress inducer Tunicamycin treatment (10 μg/mL) for 24 h (Fig. 13). The results demonstrated that exosomes from NHE7-knockdown RL95-2 cells (shNHE7 exo) significantly inhibited ER stress signaling, as shown by decreased expression of p-IRE1α, IRE1α, ATF6, GRP78, XBP1s, and CHOP (Fig. 13A). Functionally, shNHE7 exo treatment significantly suppressed cell proliferation (Fig. 13B), migration and invasion (Fig. 13C), and sphere formation (Fig. 13D) compared to control exosomes. Consistently, shNHE7 exo treatment reduced the expression of EMT-related proteins (N-cadherin and Vimentin) and stemness-related markers (OCT4, Nanog, and SOX2), while increasing E-cadherin expression (Fig. 13E). Notably, Tm treatment significantly rescued the suppressive effects of shNHE7 exo on ER stress signaling (Fig. 13A), proliferation (Fig. 13B), migration and invasion (Fig. 13C), sphere formation (Fig. 13D), as well as EMT and stemness marker expression (Fig. 13E). These results collectively indicate that NHE7 knockdown suppresses malignant phenotypes and stemness of recipient RL95-2 cells through exosome-mediated intercellular signaling transmission, and that this effect is dependent on ER stress inactivation.

### ERP27 is identified as a target of PRSS1 with expression promoted by PRSS1 in recipient EC cells

**Fig. 14:**
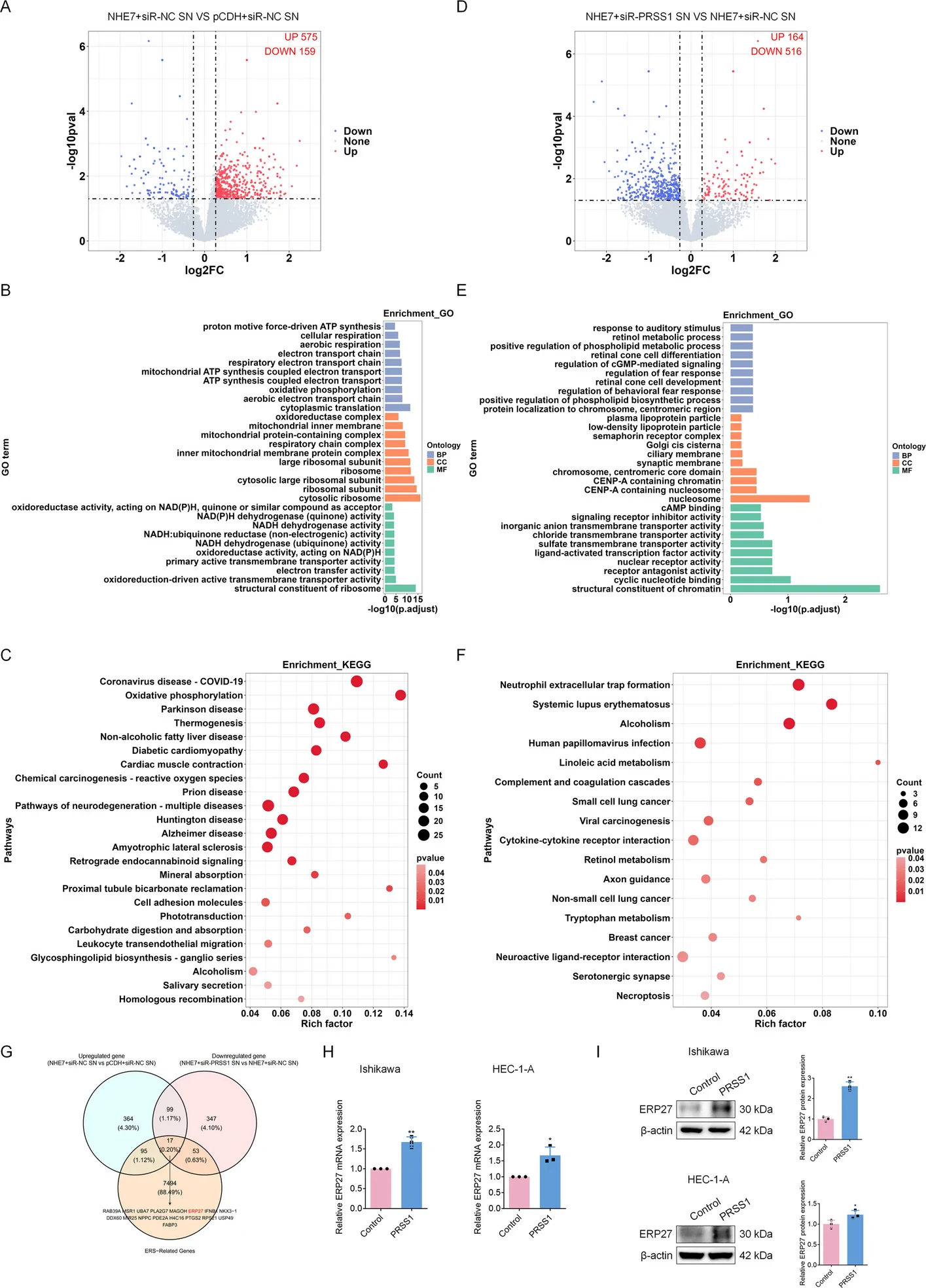
PRSS1 potentiated ER stress in recipient EC cells by upregulating ERP27 expression. Transcriptome sequencing analysis was performed to identify DEGs in Ishikawa cells incubated with supernatants from corresponding cells. **A** DEGs between Ishikawa cells incubated with supernatants from NHE7-overexpressing and control Ishikawa cells were analyzed, and their distribution was visualized using a volcano plot. **B**-**C** Functional enrichment analysis of these DEGs was performed via GO (**B**) analysis for biological processes and KEGG (**C**) analysis for signaling pathways. **D** Volcano plot analysis comparing DEGs in Ishikawa cells incubated with supernatants from NHE7-overexpressing plus PRSS1 knockdown Ishikawa cells versus those with NHE7-overexpressing alone. **E**-**F** Functional enrichment analysis of these DEGs was conducted via GO (**E**) analysis for biological processes and KEGG (**F**) analysis for signaling pathways. **G** Venn diagram showing the intersection of upregulated genes from Figure **A**, downregulated genes from Figure **D**, and ER stress-related genes. **H**-**I** Ishikawa cells were incubated with recombinant PRSS1 protein. **H** The mRNA expression level of ERP27 in Ishikawa and HEC-1-A cells was determined using qRT-PCR. **I** WB assay was performed to detect the protein expression level of ERP27 in Ishikawa and HEC-1-A cells. β-actin served as the loading control. All data were derived from at least three independent experiments. **p < 0.01, *p < 0.05 versus the control group

To investigate the intracellular mechanisms of NHE7 and PRSS1 in recipient EC cells, Ishikawa cells were treated with the corresponding cell supernatant. Comparison of transcriptomes between cells exposed to NHE7-overexpressing SN and control SN (NHE7 + siR-NC SN vs pCDH + siR-NC SN) revealed 575 upregulated DEGs and 159 downregulated DEGs (Fig. 14A). GO and KEGG enrichment analyses demonstrated predominant enrichment of these DEGs in categories related to cytoplasmic translation and oxidative phosphorylation pathways (Fig. 14B&C). Additionally, transcriptomic profiling of cells treated with corresponding cell supernatant (NHE7 + siR-PRSS1 SN vs NHE7 + siR-NC SN) identified 164 upregulated DEGs and 516 downregulated DEGs (Fig. 14D). These DEGs were predominantly associated with the regulation of cGMP-mediated signaling, Golgi cis cisterna, cAMP binding, and cytokine-cytokine receptor interaction (Fig. 14E&F).

To pinpoint PRSS1 downstream effectors, a triple intersection analysis was performed among: (1) upregulated DEGs from Fig. 14A (NHE7 + siR-NC SN vs pCDH + siR-NC SN), (2) downregulated DEGs from Fig. 14D (NHE7 + siR-PRSS1 SN vs NHE7 + siR-NC SN), and (3) ER stress-related genes from database. ERP27 was identified as a potential downstream target of PRSS1 in recipient EC cells (Fig. 14G). ERP27 (endoplasmic reticulum resident protein 27) is an ER-resident chaperone that participates in ER stress response modulation, protein folding and quality control, and trafficking [22]. Validation experiments confirmed that treatment with PRSS1 recombinant protein significantly enhanced ERP27 expression at both mRNA and protein levels in recipient EC cells (Fig. 14H&I). These results suggest that PRSS1 may potentiate ER stress in recipient EC cells by upregulating ERP27 expression, thereby influencing the malignant phenotypes of the cells.

### NHE7 upregulates c-Fos expression and enhances c-Fos binding to the PRSS1 promoter in donor EC cells via ER stress activation

**Fig. 15:**
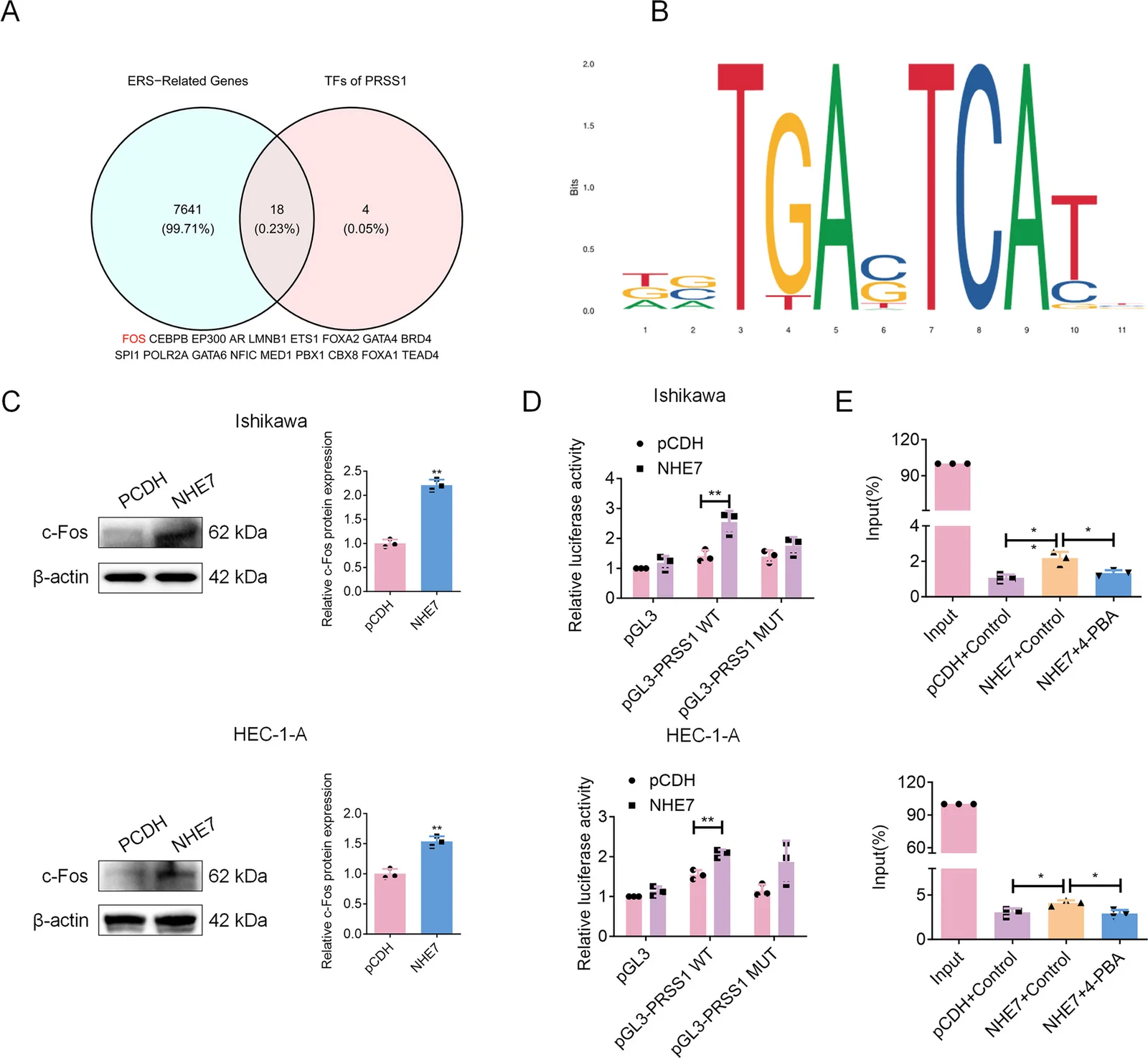
NHE7 upregulated c-Fos expression and enhanced c-Fos binding to the PRSS1 promoter in donor EC cells via ER stress activation. **A** Venn diagram was used to show the intersection between ER stress-related genes and transcription factors that can regulate PRSS1. **B** c-Fos has the putative binding sites in the promoter region of PRSS1. **C** WB assay was performed to detect the protein expression level of c-Fos in NHE7-overexpressing Ishikawa and HEC-1-A cells. **D** The potential binding site of c-Fos to the PRSS1 promoter was mutated in Ishikawa and HEC-1-A cells overexpressing NHE7. Dual-luciferase reporter assays were used to detect the luciferase intensity of the wild-type or mutant sequence. **E** The binding relationship between c-Fos and the PRSS1 promoter was verified by the ChIP assays. β-actin served as the loading control. All data were derived from at least three independent experiments. **p < 0.01, *p < 0.05 versus the control group

To further delineate the NHE7-PRSS1-centered mechanism explored in this study, we investigated the regulatory mechanism governing PRSS1 expression in donor EC cells. Based on the intersection analysis between database-curated transcription factors (TFs) capable of regulating PRSS1 and ER stress-related genes, c-Fos was identified as a key candidate regulator (Fig. 15A). c-Fos (Fos Proto-Oncogene), a pivotal transcription factor, orchestrates gene expression programs critical for stress responses, cellular damage adaptation, immune regulation, cell proliferation, and differentiation [23, 24]. Bioinformatics prediction analysis indicated potential binding sites for c-Fos within the PRSS1 promoter region (Fig. 15B). Notably, NHE7 overexpression enhanced c-Fos protein expression (Fig. 15C), indicating a mechanistic link wherein NHE7 upregulates c-Fos expression to facilitate its binding to the PRSS1 promoter.

To validate this hypothesis, we generated luciferase reporter constructs bearing either the wild-type PRSS1 promoter or a mutant promoter with disrupted c-Fos binding sites. NHE7 overexpression significantly increased luciferase activity driven by the PRSS1-WT promoter, while exhibiting no effect on the mutant promoter (Fig. 15D). Importantly, ChIP assays using an anti-c-Fos antibody confirmed enhanced recruitment of c-Fos to the PRSS1 promoter in NHE7-overexpressing cells, an interaction that was attenuated by treatment with the ER stress inhibitor 4-PBA (Fig. 15E). Collectively, these findings demonstrate that in donor EC cells, NHE7 activates ER stress to upregulate c-Fos expression, which enhances c-Fos binding to the PRSS1 promoter and drives its transcription.

### NHE7 promotes EC tumor growth in vivo through ER stress activation

**Fig. 16:**
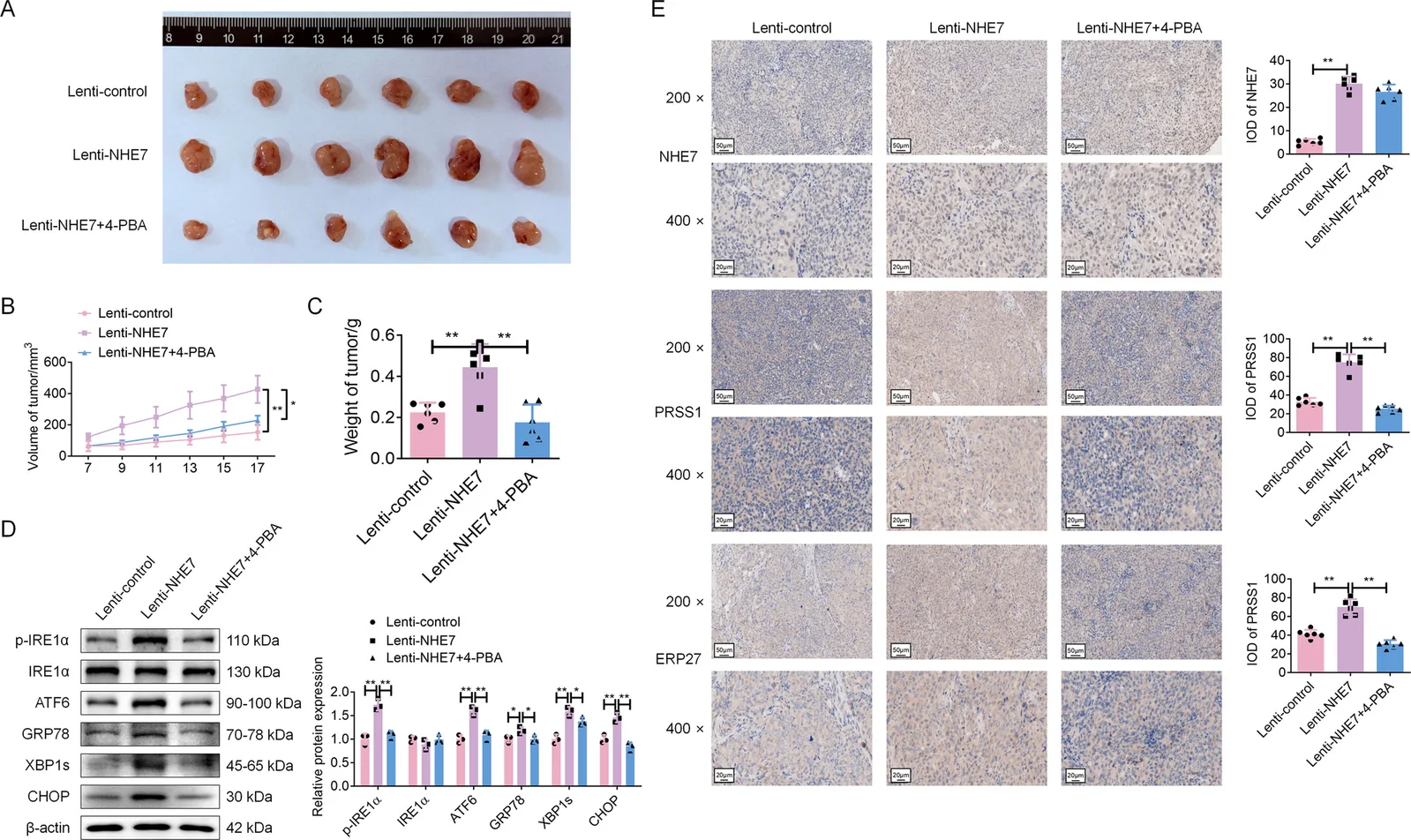
NHE7 promoted EC tumor growth in vivo through ER stress activation. The nude mice (*n* = 6) were injected with Ishikawa cells overexpressing NHE7 to establish tumor xenograft models. The mice were intraperitoneally injected with 4-PBA solution (150 mg/kg) for four consecutive days. **A** The xenograft tumors were isolated from the mice. **B** Tumor growth was recorded every two days from day 7 to day 17. **C** The weights of the tumors were recorded. **D** WB assay was used to detect the expression of ER stress-related proteins in the tumor tissues. **E** The expression levels of NHE7, PRSS1, and ERP27 in the tumor tissues were examined by IHC assay. β-actin served as the loading control. **p < 0.01, *p < 0.05 versus the control group

The mice were randomly divided into three groups (*n* = 6 per group) and subcutaneously injected with Ishikawa cells stably overexpressing NHE7 or control vector. For the model group, a mixture (1:1) of 2 × 10^6^ NHE7-overexpressing Ishikawa cells and 2 × 10^6^ parental Ishikawa cells was injected, while the control group received 4 × 10^6^ parental Ishikawa cells alone. The mice were intraperitoneally injected with 4-PBA solution (150 mg/kg) for four consecutive days. Tumor growth was closely monitored, with volumes measured at two-day intervals. Once tumors reached approximately 1500 mm^3^, mice were euthanized, and tumors were harvested for volume and weight determination. NHE7 overexpression significantly accelerated EC tumor growth, while 4-PBA treatment markedly attenuated this pro-tumorigenic effect (Fig. 16A-C). WB analysis confirmed that 4-PBA treatment suppressed NHE7-induced ER stress activation in tumor tissues (Fig. 16D). Consistent with in vitro findings, IHC analysis demonstrated that NHE7 overexpression led to elevated levels of PRSS1 and ERP27 in tumor tissues, an effect that was reversed by 4-PBA treatment (Fig. 16E). Collectively, these in vivo data support the conclusion that NHE7 promotes PRSS1 expression through ER stress activation, thereby enhancing EC tumor progression.

## Discussion

This study delineates a sophisticated signaling cascade in which NHE7 orchestrates endometrial cancer progression through ER stress-mediated transcriptional reprogramming, paracrine signaling, and exosomal communication. Our findings establish that NHE7 functions as a key regulator of ER stress in EC cells, driving malignant phenotypes and cellular stemness through PRSS1-dependent intercellular communication.

Specifically, in donor EC cells, NHE7 upregulates c-Fos expression and facilitates its binding to the PRSS1 promoter via ER stress activation, thereby driving the transcription and expression of PRSS1. In recipient EC cells, secreted PRSS1 acts as a paracrine effector by upregulating ERP27 expression, thereby amplifying ER stress responses. This intercellular communication network creates a feed-forward loop that sustained ER stress activation in the tumor microenvironment. Collectively, these processes enhanced malignant phenotypes and stem-like characteristics in EC cells through dual mechanisms: (1) autocrine ER stress activation in donor cells, and (2) paracrine ER stress propagation via PRSS1-ERP27 signaling in recipient cells. In vivo studies confirmed that the NHE7-PRSS1-ERP27 axis accelerates tumor growth by maintaining chronic ER stress, providing a novel therapeutic target for endometrial cancer intervention.

The Na^+^/H^+^ exchanger family has recently emerged as an important regulator of intracellular pH homeostasis in cancer cells, with several isoforms implicated in tumor cell survival, metabolism, and metastasis. Among them, the role of NHE7 in cancer has increasingly attracted research attention, particularly concerning tumor cell proliferation, survival, and metastasis [4, 25, 26]. Consistent with previous studies that explored the functions of NHE7 in other cancer types, our results demonstrate that NHE7 overexpression significantly enhances cell proliferation, migration, invasion, and self-renewal capabilities in EC cells, while NHE7 knockdown suppresses these malignant phenotypes (Figs. 1 and 3). A key insight from our work is that NHE7 triggers the unfolded protein response (UPR) and endoplasmic reticulum (ER) stress pathways. ER stress is characterized by the activation of three canonical transducers: PERK (protein kinase RNA-like ER kinase), IRE1α (inositol-requiring enzyme 1 alpha), and ATF6 (activating transcription factor 6). Downstream markers include GRP78 (glucose-regulated protein 78), XBP1s (X-box binding protein 1 spliced), and CHOP (C/EBP homologous protein), which collectively coordinate adaptive responses to restore ER homeostasis [27–29]. Transcriptomic analysis revealed significant enrichment of ER stress-related DEGs upon NHE7 overexpression, and western blotting confirmed phosphorylation of PERK and IRE1α, as well as upregulation of ATF6, GRP78, XBP1s, and CHOP (Fig. 2). Conversely, NHE7 knockdown suppressed these ER stress markers (Fig. 3).

This is mechanistically significant given ER stress’s established role in tumor cell proliferation and survival, angiogenesis, metastasis, and immune evasion [8, 30]. For example, PERK/eIF2α and IRE1/XBP1 pathways can promote cancer cell survival under hypoxia and nutrient stress, and can drive EMT and migration through downstream effectors [31, 32]. It has been reported that ER stress signaling is associated with the maintenance of cancer cell plasticity, including EMT and cancer stem cell (CSC) properties [33, 34]. In our experiments, NHE7-induced ER stress correlated with downregulation of E-cadherin and upregulation of N-cadherin and Vimentin, classical EMT markers, supporting this connection. Thus, NHE7-driven ER stress likely contributed to the EMT process and enhanced stemness we observed.

A central focus of this study was the exploration of intercellular signaling dynamics mediated by ER stress. We found that conditioned medium (supernatant) from NHE7-overexpressing EC cells could transfer malignant traits and ER stress activation to naïve EC recipient cells. Recipient cells exposed to NHE7-supernatant exhibited increased proliferation, migration, invasion, sphere-forming capacity, and expression of stemness markers (OCT4, Nanog, and SOX2) compared to controls (Fig. 4). NHE7-supernatant also activated ER stress signaling in EC recipient cells, as evidenced by increased expression of p-IRE1α, IRE1α, ATF6, GRP78, XBP1s, and CHOP (Fig. 4). Conversely, supernatant from NHE7-knockdown RL95-2 cells suppressed these malignant phenotypes and ER stress activation in recipient cells (Fig. 5). This finding suggests that NHE7 can influence neighboring cells through the secretion of factors that activate ER stress signaling pathways. Notably, treatment with 4-PBA, an ER stress inhibitor, markedly attenuated these effects, reinforcing the evidence that ER stress is a critical mediator of NHE7-driven intercellular signaling (Fig. 6).

Crucially, the investigation of PRSS1, identified as a pivotal downstream target of NHE7, provides further insight into the mechanisms by which NHE7 influences malignant behavior in EC cells. The exosome inhibitor GW4869 was used to confirm that NHE7 transmits signals through exosomes. GW4869 treatment of donor NHE7-overexpressing cells significantly attenuated NHE7 supernatant-induced malignant phenotypes and ER stress activation in recipient cells (Fig. 7). Bioinformatic overlap of NHE7-responsive genes with ER stress-related genes highlighted several candidates, of which PRSS1 was the most significant upregulation induced by NHE7. Consistently, NHE7 overexpression significantly elevated mRNA and protein expression of PRSS1 in EC cells (Fig. 8). PRSS1 knockdown in RL95-2 cells confirmed that PRSS1 is a downstream target of NHE7, as evidenced by reduced PRSS1 expression at both mRNA and protein levels, as well as decreased PRSS1 levels in supernatant and exosomes (Fig. 9). Direct treatment of recipient EC cells with recombinant PRSS1 protein induced ER stress activation (as shown by increased p-IRE1α, IRE1α, ATF6, GRP78, XBP1s, and CHOP), enhanced proliferation, migration, invasion, sphere formation, EMT, and stemness (Fig. 10). To further validate that PRSS1 mediates NHE7-driven intercellular signaling, PRSS1 was knocked down in donor cells. The supernatant from NHE7-overexpressing donor cells with or without PRSS1 knockdown was collected and added to recipient cells. The results showed that PRSS1 knockdown significantly attenuated NHE7 supernatant-induced malignant phenotypes and ER stress activation in recipient cells (Fig. 11). Moreover, exosomes directly isolated from NHE7-overexpressing donor cells were sufficient to induce malignant phenotypes and ER stress in recipient cells, and this effect was reduced when PRSS1 was knocked down in donor cells (Fig. 12). Conversely, exosomes from NHE7-knockdown RL95-2 cells suppressed malignant phenotypes and ER stress in recipient cells, and treatment with the ER stress inducer Tunicamycin rescued these suppressive effects (Fig. 13). Exosomes and other extracellular vesicles are known to mediate intercellular communication in tumors, often carrying stress signals and proteins that remodel the tumor microenvironment [35–37]. Moreover, ER stress itself can promote secretion of exosomes enriched in oncogenic cargo [38–40]. Our data showed that supernatant and exosomes derived from NHE7-overexpressing cells were enriched for PRSS1, suggesting that PRSS1 may serve as a paracrine factor, capable of modulating the behavior of surrounding cells within the tumor microenvironment. IHC staining of clinical EC samples revealed a positive correlation between NHE7 and PRSS1 expression at the tissue level, and bioinformatics analysis further supported their potential functional association in EC progression (Fig. 8&S2).

PRSS1 encodes the precursor of trypsin, a secreted serine protease. Although PRSS1 is best known for its role in the pancreas, its expression has been documented in various tumors [21, 41–43]. Functionally, PRSS1 overexpression has been shown to enhance tumor cell growth and invasion. For instance, PRSS1 promoted proliferation in gastric carcinoma cells, whereas its silencing inhibited proliferation and metastasis through downregulation of ERK signaling [42]. In our work, the data reveal its novel function in mediating ER stress transmission in EC.

Our exploration of the downstream effects of PRSS1 in recipient cells revealed ERP27 as a key target. Using triple intersection analysis of transcriptomic data from recipient EC cells treated with different supernatants, ERP27 was identified as a potential downstream target of PRSS1 (Fig. 14). Validation experiments confirmed that recombinant PRSS1 treatment significantly enhanced ERP27 expression at both mRNA and protein levels in recipient EC cells (Fig. 14). The role of ERP27 as a chaperone in the ER stress response adds another layer to our understanding of NHE7's involvement in EC progression, as it suggests that NHE7 might influence protein folding and trafficking within the ER, thereby impacting tumor biology. ERP27 is a non-catalytic member of the protein disulfide isomerase (PDI) family and an ER-resident chaperone that modulates UPR magnitude and ensures protein folding fidelity [16, 44, 45]. PDI family proteins are frequently overexpressed in cancers and often contribute to malignancy. High expression of various PDIs supports tumor cell survival, progression, and metastasis [46]. For example, ERP29 (another non-catalytic PDI) has been shown to promote EMT and invasion in cancer [46, 47]. In this study, recombinant PRSS1 elevated the mRNA and protein expression of ERP27 in recipient EC cells, suggesting that ERP27 upregulation amplifies ER stress signaling.

To understand how NHE7 induces PRSS1 transcription, we intersected ER stress-related transcription factors with PRSS1 regulators, identifying AP-1 transcription factor c-Fos as a central node. NHE7 overexpression increased c-Fos levels and promoted the binding of c-Fos to the PRSS1 promoter (Fig. 15). This is consistent with recent reports that link AP-1 family transcription factors to UPR-driven gene expression [48–50]. Crucially, treatment with 4-PBA reduced c-Fos binding to the PRSS1 promoter, indicating that NHE7-induced ER stress is required for c-Fos activation of PRSS1 (Fig. 15). This creates a feed-forward loop that NHE7 upregulates c-Fos expression by triggering ER stress; c-Fos then drives PRSS1 transcription, leading to more secreted PRSS1 and propagation of ER stress. This mechanism illustrates how ER stress can reprogram transcriptional programs to sustain oncogenic signaling, emphasizing the interplay between UPR and gene regulation.

The in vivo relevance of this axis was further validated using a mouse xenograft model. NHE7 overexpression significantly accelerated EC tumor growth, while treatment with the ER stress inhibitor 4-PBA markedly attenuated this pro-tumorigenic effect (Fig. 16). WB analysis of tumor tissues confirmed that 4-PBA suppressed NHE7-induced ER stress activation, and IHC analysis demonstrated that NHE7 overexpression led to elevated levels of PRSS1 and ERP27 in tumor tissues, effects that were reversed by 4-PBA treatment (Fig. 16). These in vivo data further support the conclusion that the NHE7-PRSS1-ERP27 axis promotes EC tumor progression through ER stress activation.

In summary, this study demonstrates that NHE7 promotes EC progression by activating ER stress, which upregulates c-Fos to drive PRSS1 transcription in donor cells. PRSS1 is then secreted via exosomes and acts on recipient cells to upregulate ERP27, amplifying ER stress and promoting malignant phenotypes including proliferation, migration, invasion, EMT, and stemness.

Although this study provides significant insights into the role of the NHE7-PRSS1-ERP27 axis in EC, several limitations merit consideration. Firstly, the receptors or uptake mechanisms by which extracellular PRSS1 activates UPR in recipient cells are undefined, and candidates include protease-activated receptors or endocytic pathways. Secondly, the spectrum of cargo in NHE7-driven exosomes beyond PRSS1 warrants proteomic profiling. Thirdly, we have not examined NHE7 function in non-cancerous endometrial epithelial cells (such as hEEC, T-HESC, or primary normal endometrial epithelial cells). Notably, EC cell lines exhibit varying basal levels of NHE7 expression, and RL95-2 cells with high endogenous NHE7 display elevated ER stress compared to Ishikawa and HEC-1-A cells. Whether the NHE7-ER stress-PRSS1 axis operates specifically in the malignant context or represents a more general cellular response remains unknown. Fourth, regarding the role of exosomes in NHE7-mediated intercellular signaling, our study relied primarily on electron microscopy images to identify exosomes and on GW4869 to provide functional evidence. While these data support the involvement of exosomes, GW4869 provides functional rather than direct biochemical evidence for increased exosome release. Additionally, our exosome isolation by ultracentrifugation may have limitations regarding co-isolated contaminants. Fifth, the precise mechanisms by which NHE7 overexpression induces ER stress remain to be distinguished. Possible mechanisms include crowding of the Golgi apparatus causing retrograde trafficking of proteins from the Golgi back to the ER, or direct protein spillover into the ER. Sixth, while our recombinant PRSS1 protein and PRSS1 knockdown experiments demonstrate that PRSS1 is a key mediator of NHE7 SN effects, neutralizing antibodies against PRSS1 would provide more direct evidence by blocking its activity in the supernatant. Seventh, while we used 4-PBA as a chemical chaperone to inhibit ER stress, it should be noted that 4-PBA has been reported to have off-target effects, including HDAC inhibitor activity and mRNA translation inhibition, which may contribute to its anti-cancer effects independently of ER stress modulation.

Future studies using non-cancerous cell lines will be necessary to determine the context-dependent functions of NHE7. Direct quantification methods such as nanoparticle tracking analysis will be needed to further characterize the effect of NHE7 on exosome biogenesis and release. Complementary purification methods for exosomes, such as size exclusion chromatography, density gradient centrifugation, or immunomagnetic beads, should be employed in future studies to obtain purer exosome preparations and validate the enrichment of PRSS1. The precise mechanisms by which NHE7 overexpression induces ER stress, whether through crowding of the Golgi apparatus causing retrograde trafficking or direct protein spillover into the ER, remain to be distinguished. The use of neutralizing antibodies against PRSS1 would provide more direct evidence that PRSS1 specifically mediates NHE7 effects. Genetic approaches (e.g., CRISPR-mediated knockdown of key UPR components such as PERK, IRE1α, or ATF6) or more specific ER stress inhibitors will be necessary to confirm the specific role of ER stress in NHE7-mediated EC progression without the confounding effects associated with pharmacological inhibitors like 4-PBA. Finally, whether NHE7-PRSS1 signaling interacts with immunomodulatory pathways represents an important avenue for future exploration, given emerging roles for ER stress in shaping tumor immune landscapes.

## Conclusion

In conclusion, our work delineates a novel NHE7-PRSS1-ERP27 axis that propagates ER stress signaling to drive EC progression. This study illustrates the multifaceted roles of NHE7 in EC progression, highlighting its ability to activate ER stress signaling, promote PRSS1 expression and secretion, and enhance malignant phenotypes and cellular stemness in EC cells. The findings open new avenues for understanding how NHE7 and ER stress signaling transmission can impact tumor biology and suggest potential targets for therapeutic intervention in EC and possibly other malignancies. Future investigations should focus on delineating the precise signaling interactions engaged by NHE7 and PRSS1, along with exploring the potential of PRSS1 as a biomarker for EC prognosis.

## Supplementary Information

Supplementary Material 1: Figure S1. Survival analysis of NHE7-regulated ER stress-related genes in TCGA endometrial cancer dataset. (A) Kaplan–Meier survival analysis of the 9 intersecting genes was performed using TCGA endometrial cancer dataset.

Supplementary Material 2: Figure S2. PRSS1 was highly expressed and associated with a poor prognosis in EC. (A) Based on the median expression of PRSS1, EC samples were categorized into two groups: a high PRSS1 expression group and a low PRSS1 expression group. The DESeq2 R Package version 1.46.0 generated the volcano plot and showed the distribution of DEGs. The x-axis is log2 of fold change and the y-axis is the *P* value (-log10). (B-C) TCGA database was used to analyze (B) the prognostic analysis of PRSS1 expression (C) and the expression of PRSS1 in tumor tissues and adjacent normal tissues in EC. (D) Correlation analysis between PRSS1 and SLC9A7 (NHE7) expression using the same set of TCGA tumor samples (*n* = 550). Scatter plot was generated with the ggpubr package.

Supplementary Material 3: Figure S3. Knockdown efficiency of PRSS1 in EC cells. (A) PRSS1 was knocked down in Ishikawa cells via transient transfection, and the transfection efficiency was determined by WB assays. β-actin served as the loading control. **p < 0.01, *p < 0.05 versus the control group.

## Data Availability

All data generated or analyzed during this study are shown in this published article.
